# At the confluence of vicariance and dispersal: Phylogeography of cavernicolous springtails (Collembola: Arrhopalitidae, Tomoceridae) codistributed across a geologically complex karst landscape in Illinois and Missouri

**DOI:** 10.1002/ece3.4507

**Published:** 2018-09-24

**Authors:** Aron D. Katz, Steven J. Taylor, Mark A. Davis

**Affiliations:** ^1^ Department of Entomology University of Illinois at Urbana‐Champaign Urbana Illinois USA; ^2^ Illinois Natural History Survey Prairie Research Institute University of Illinois at Urbana‐Champaign Champaign Illinois USA; ^3^ Present address: Office of General Studies Colorado College Colorado Springs Colorado USA

**Keywords:** biospeleology, cryptic diversity, ecology, evolution, Mississippi River, short‐range endemism

## Abstract

The processes of vicariance and dispersal are central to our understanding of diversification, yet determining the factors that influence these processes remains a significant challenge in evolutionary biology. Caves offer ideal systems for examining the mechanisms underlying isolation, divergence, and speciation. Intrinsic ecological differences among cavernicolous organisms, such as the degree of cave dependence, are thought to be major factors influencing patterns of genetic isolation in caves. Using a comparative phylogeographic approach, we employed mitochondrial and nuclear markers to assess the evolutionary history of two ecologically distinct groups of terrestrial cave‐dwelling springtails (Collembola) in the genera *Pygmarrhopalites* (Arrhopalitidae) and *Pogonognathellus* (Tomoceridae) that are codistributed in caves throughout the Salem Plateau—a once continuous karst region, now bisected by the Mississippi River Valley in Illinois and Missouri. Contrasting phylogeographic patterns recovered for troglobiotic *Pygmarrhopalites* sp. and eutroglophilic *Pogonognathellus* sp. suggests that obligate associations with cave habitats can restrict dispersal across major geographic barriers such as rivers and valleys, but may also facilitate subterranean dispersal between neighboring cave systems. *Pygmarrhopalites* sp. populations spanning the Mississippi River Valley were estimated to have diverged 2.9–4.8 Ma, which we attribute to vicariance resulting from climatic and geological processes involved in Mississippi River Valley formation beginning during the late Pliocene/early Pleistocene. Lastly, we conclude that the detection of many deeply divergent, morphologically cryptic, and microendemic lineages highlights our poor understanding of microarthropod diversity in caves and exposes potential conservation concerns.

## 1 INTRODUCTION

Subterranean ecosystems are attractive systems for biologists seeking to understand the evolutionary processes that shape patterns of biological diversity (Culver & Pipan, 2009a). These isolated, dark, low‐energy habitats promote adaptation and diversification (Ortiz et al., 2014) and provide the ecological context for examining mechanisms underlying divergence and speciation (e.g., Gómez, Reddell, Will, & Moore, 2016; Juan, Guzik, Jaume, & Cooper, 2010; Niemiller, Fitzpatrick, & Miller, 2008; Schonhöfer, Vernesi, Martens, & Marshal, 2015). In contrast to their surface relatives, many cave‐dwelling species have morphological, physiological, and behavioral adaptations that limit or prevent surface dispersal (White & Culver, 2012). Often restricted to small, discontinuous ranges, these species can exhibit high levels of population structure, short‐range endemism, and morphologically cryptic species (e.g., Christman, Culver, Madden, & White, 2005; Faille, Tänzler, & Toussaint, 2015; Niemiller, Near, & Fitzpatrick, 2012; Zagmajster, Culver, & Sket, 2008).

Genetic isolation is a primary driver of molecular divergence and ultimately speciation, but determining the factors that promote or constrain genetic diversity remains a significant challenge in evolutionary biology. Patterns of diversity in caves are often attributed to vicariance or dispersal, but the relative influence these processes have on the evolution and contemporary distributions of cave fauna has been widely debated (see Culver, Pipan, & Schneider, 2009; Porter, 2007). However, it is generally accepted that patterns of diversity in caves are likely shaped by a complex interaction of intrinsic factors (e.g., species‐specific differences in ecology, life history, or biology) that can influence dispersal capacity and extrinsic factors (e.g., geographic barriers or climate change) that can enhance or limit dispersal opportunity (Juan et al., 2010; Porter, 2007).

Phylogeography, the study of processes that influence the contemporary geographic distributions of species’ populations by utilizing genetic data can provide insights into the relative influences of evolutionary factors driving patterns of genetic isolation and divergence in biological communities (Avise, 2000; Avise et al., 1987). For instance, phylogeographic congruence among codistributed species can implicate vicariance caused by “hard” geographic barriers or environmental changes affecting entire communities (Lapointe & Rissler, 2005), whereas conflicting phylogeographic patterns may be attributable to intrinsic differences that can affect species dispersal capacity across “soft” potential genetic barriers (e.g., Goldberg & Trewick, 2011; Hodges, Rowell, & Keogh, 2007; Hurtado, Lee, & Mateos, 2013). With cave organisms, the majority of research studies have been limited to single species (e.g., Dörge, Zaenker, Klussmann‐Kolb, & Weigand, 2014; Faille et al., 2015) or cryptic species complexes with allopatric distributions (e.g., Gómez et al., 2016; Rastorgueff, Chevaldonné, Arslan, Verna, & Lejeusne, 2014). Few studies have incorporated phylogeographic approaches that consider intrinsic differences among codistributed cave‐dwelling species (see Pérez‐Moreno, Balázs, Wilkins, Herczeg, & Bracken‐Grissom, 2017; Weckstein et al., 2016).

The arthropod class Collembola (springtails) offers a nearly unparalleled opportunity for elucidating the interplay of factors that affect speciation and molecular diversification in subterranean ecosystems. These small, wingless, insect‐like arthropods are among the most abundant, diverse, and well‐adapted organisms in caves (Christiansen, 1965; Thibaud & Deharveng, 1994), and are considered important subterranean examples of adaptive radiations (Christiansen & Culver, 1969) and parallel speciation (Christiansen, 1961, 1965; Christiansen & Culver, 1968). Their small size (body length often less than 1 mm), low vagility, and close associations with cave habitats facilitate their isolation, resulting in a high degree of endemism (Niemiller & Zigler, 2013) and cryptic species (Juan & Emerson, 2010). For example, the springtail genus *Pseudosinella* alone contains more than 100 species found in caves worldwide, many of which are known only from a single cave system (Hopkin, 1997). Most importantly, cave‐dwelling springtails have varying levels of ecological specificity to, and dependence upon, cave habitats. Although surface species are commonly found in caves as accidentals (i.e., they may fall or get washed into caves, but cannot maintain populations in caves), the majority of collembolans occurring in caves can maintain permanent subterranean populations and are either classified as troglobionts (i.e., obligate cave‐dwellers that are never encountered on the surface and often have conspicuous troglomorphic adaptations associated with cave habitats) or eutroglophiles (i.e., facultative cave‐dwellers that also occur in surface habitat and usually lack apparent troglomorphy) (see Sket, 2008 for current ecological classifications of subterranean animals). Because troglobiotic and eutroglophilic springtails can be codistributed (Katz et al., 2016; Soto‐Adames & Taylor, 2013), extrinsic evolutionary processes are likely exerting similar selective pressures upon them. Therefore, opposing patterns of genetic structure among these species distributed across the same geographic area can reflect intrinsic factors, such as differences in the degree of ecological association with cave habitats (cave dependence) that can affect a species’ capacity to disperse across geographic barriers (Pérez‐Moreno et al., 2017; Weckstein et al., 2016). Disparate geographic distributions among closely related surface springtails provide some indirect evidence that varying dispersal capacity may be associated with differences in species‐specific traits (Costa et al., 2013; Katz, Giordano, & Soto‐Adames, 2015), and Christiansen and Culver's (1987) biogeographic study of cave springtails revealed that more pronounced troglomorphy can be correlated with smaller geographic ranges.

Long‐term local persistence and small geographic ranges are typical for troglobionts, and by definition, these species cannot maintain surface populations to facilitate dispersal between discontinuous subterranean habitats. Therefore, patterns of genetic differentiation in troglobionts are likely driven primarily by isolation due to physical barriers and reflect vicariance. On the contrary, we expect isolation by distance (IBD) to be the primary driver of genetic variation in eutroglophile*s* owing to their propensity to disperse across surface habitats.

To test these predictions, we incorporate a suite of molecular‐based approaches to (a) delimit cryptic species in the focal complexes, (b) detect molecular signatures of isolation to identify potential genetic barriers, and (c) estimate evolutionary relationships and divergence times to elucidate the roles of vicariance and dispersal in shaping patterns of cave‐dwelling springtail diversity throughout the Salem Plateau—a major cave‐bearing karst region of the Ozark Plateau that spans the Mississippi River Valley in Illinois and Missouri. Recent molecular‐based biogeographic investigations of Ozark cave biodiversity have been useful for addressing evolutionary hypotheses for salamanders (Phillips, Fenolio, Emel, & Bonett, 2017) and fish broadly distributed across the Mississippi River Valley (Niemiller et al., 2012). However, the phylogeography of cave invertebrates has yet to be evaluated for the Salem Plateau. Fine‐scale phylogeographic patterns of cave springtails distributed across the Mississippi River may be used to investigate the impact of intrinsic and extrinsic factors (e.g., the degree of cave dependence and geographic barriers) on the evolution of cave organisms, broaden our limited understanding of subterranean microarthropod diversity, and assess biogeographic interpretations that may help clarify the complex, yet poorly understood, geological history of the Salem Plateau.

## 2 MATERIALS AND METHODS

### 2.1 Study system, focal taxa, and field collections

**Figure 1:**
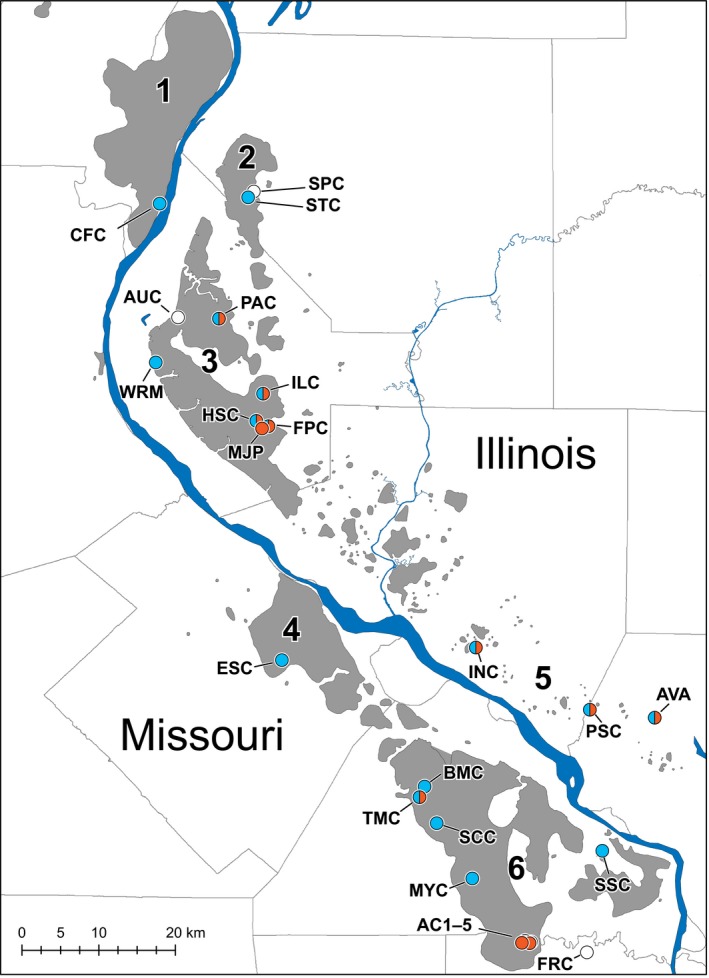
Salem Plateau cave‐bearing karst spanning the Mississippi River border of Illinois and Missouri (gray) (adapted from Panno et al. (1997, 1999)). Sinkhole karst areas labeled 1–6; Colored dots indicate the presence/absence of focal taxa at each sampled cave (*Pygmarrhopalites* present, blue; *Pogonognathellus* present, red; both taxa absent, white). Cave name abbreviations are listed in Table 1

**Figure 2:**
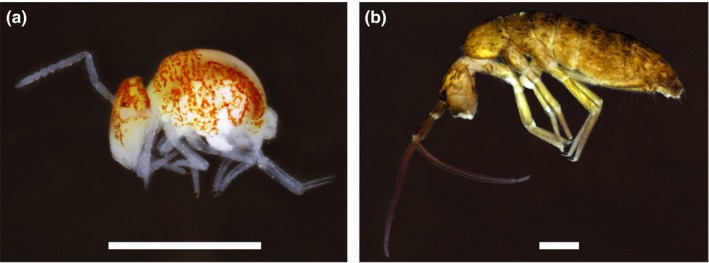
Photographs of (a) *Pygmarrhopalites* sp. collected from Pautler Cave in Illinois and (b) *Pogonognathellus* pale species complex collected from Fogelpole Cave in Illinois. Scale bars = 500 μm

The complex geological landscape of the Salem Plateau (Figure 1) provides the ecological context for testing biogeographic hypotheses of vicariance and dispersal. This once continuous karst region, now bisected by the Mississippi River Valley, is located south of St. Louis and covers just eight counties but contains thousands of sinkholes and includes the largest cave systems in Illinois and Missouri (Panno, Weibel, & Li, 1997). Here, we examine patterns of molecular diversity of two codistributed and ecologically distinct genera of springtails—*Pygmarrhopalites* Vargovitch, 2009 (Arrhopalitidae) and *Pogonognathellus* Paclt, 1944 (Tomoceridae) (Figure 2)—both highly abundant groups that likely comprise the majority of cave‐dwelling Collembola found in this region. Most *Pygmarrhopalites* species in caves are classified as troglobionts because they are usually troglomorphic and have never been reported in surface habitats, instead occurring in the dark zone of caves, primarily as epipleuston upon standing water surfaces (e.g., drip pools) or in accumulations of organic debris. In contrast, *Pogonognathellus* species are not troglobionts and include only a few eutroglophilic species that can maintain permanent populations in caves. Eutroglophilic *Pogonognathellus* occur in both surface and cave habitats, often in abundance on organic debris and rock surfaces in cave entrances and twilight zones, and less frequently and in smaller numbers in cave dark zones.

To date, 12 species of *Pygmarrhopalites* and four species of *Pogonognathellus* have been reported in Salem Plateau caves (Lewis, Moss, Tecic, & Nelson, 2003; Peck & Lewis, 1978; Soto‐Adames & Taylor, 2010, 2013; Zeppelini & Christiansen, 2003), including nine species of *Pygmarrhopalites* that are classified as troglobionts and a single, but widespread, eutroglophilic species complex—the *Pogonognathellus* pale complex (Felderhoff, Bernard, & Moulton, 2010), formerly recognized as Nearctic populations of *Pogonognathellus flavescens* (Tullberg, 1871).

Invertebrate surveys were conducted in 25 caves located throughout the Salem Plateau karst in Illinois and Missouri during the summer of 2016 (Table 1; Figure 1). At each cave site, the dominant habitat types where springtails are known to occur were opportunistically sampled from cave entrance, twilight, and dark zones. Habitats such as rock surfaces, scat, and drip pools were sampled manually using an aspirator. Invertebrates were also extracted from organic debris (collected into bags at each cave) using Berlese funnels. Ecological data, such as sample orientation (wall, floor), substrate, habitat, and cave zone, were recorded for each sample. All Collembola recovered from raw samples were subsequently sorted by morphospecies (i.e., groups of individuals which are morphologically indistinguishable under stereomicroscopy, having similar color pattern, appendage proportions, and body shape) using a Leica MZ12.5 research stereomicroscope. The most abundant morphospecies of *Pygmarrhopalites* and *Pogonognathellus* were retained for DNA extraction. All sorted samples, including nontarget specimens, were stored in 95% EtOH at 4°C.

**Table 1:** Caves sampled for this study including abbreviated cave names, locality information, and number of *Pygmarrhopalites* (A) and *Pogonognathellus* (T) specimens sequenced from each cave

Cave	Cave abbrev.	State	County	Sinkhole area^a^	A(*n*)	T(*n*)
Ava Cave	AVA	IL	Jackson	5	1	5
Polystichum acrostichoides Sink Cave	PSC	IL	Jackson	5	1	1
Auctioneer Cave	AUC	IL	Monroe	3	0	0
Fogelpole Cave	FPC	IL	Monroe	3	6	8
Hoppy Speck Cave	HSC	IL	Monroe	3	4	3
Illinois Caverns	ILC	IL	Monroe	3	1	2
Mead Jars Pit	MJP	IL	Monroe	3	0	1
Pautler Cave	PAC	IL	Monroe	3	4	1
White Rock Mine	WRM	IL	Monroe	3	1	0
Indian Cave	INC	IL	Randolph	5	2	1
Sphalloplana Stream Cave	SPC	IL	St. Clair	2	0	0
Stemmler Cave	STC	IL	St. Clair	2	1	0
Apple Creek 1	AC1	MO	Cape Girardeau	6	0	1
Apple Creek 3	AC3	MO	Cape Girardeau	6	0	1
Apple Creek 4	AC4	MO	Cape Girardeau	6	0	0
Apple Creek 5	AC5	MO	Cape Girardeau	6	0	0
Flat Rock Creek Cave	FRC	MO	Cape Girardeau	6	0	0
Apple Creek 2	AC2	MO	Perry	6	0	0
Berome Moore Cave	BMC	MO	Perry	6	5	0
Mystery Cave	MYC	MO	Perry	6	4	0
Seventy‐six Cave	SSC	MO	Perry	6	8	0
Streiler City Cave	SCC	MO	Perry	6	1	0
Tom Moore Cave	TMC	MO	Perry	6	1	1
Cliff Cave	CFC	MO	St. Louis	1	1	0
Esoteric Cave	ESC	MO	Ste. Genevieve	4	1	0

*Notes*

Refers to high‐density sinkhole areas in the Salem Plateau karst study area defined for Illinois (Panno et al., 1997, 1999; Venarsky et al., 2009) and Missouri (Burr et al., 2001; Panno et al., 1999) (see Figure 1).

Because caves contain sensitive resources, including federally endangered species, specific locations are not included in supporting material—these data must be requested from the Illinois Speleological Survey or the Missouri Speleological Survey.

### 2.2 DNA extraction, PCR amplification, DNA sequencing, and alignment

Chaetotaxy (i.e., the arrangement and morphology of setae) and other small cuticular morphological characters are critical for springtail species identification, and it is often impossible to identify springtails to the species‐level without first making slides—a process that destroys DNA. Therefore, DNA was extracted from specimens representing the most abundant morphospecies for *Pygmarrhopalites* (*n* = 43) and *Pogonognathellus* (*n* = 26) using the following modifications to the DNeasy Blood & Tissue kit protocol for maximizing DNA concentrations while preserving morphology to associate genetic sequences with voucher specimens for species identifications (Qiagen Inc., Valencia, CA, USA): (a) Specimens were incubated overnight at 56°C after the addition of ATL buffer with proteinase‐K; (b) after the addition of EtOH, the samples were stored at 4°C overnight in order to maximize DNA precipitation; and (c) prior to centrifugation, buffer containing DNA was carefully removed and added to a column using a pipette, taking care to not lose or damage the specimens which were left at the bottom of the tube and preserved in 95% EtOH. Digestion of tissues and pigments by the lysis buffer resulted in very delicate and clear specimens, ready for slide mounting without additional preparation, but the fragile cuticles were easily damaged when handled and small individuals were nearly invisible making them difficult to recover. Therefore, the heads of specimens, which include important diagnostic morphology (e.g., the arrangement and morphology of setae), were dissected and stored separately prior to DNA extraction as back up vouchers for those cases where the now‐translucent bodies were not recovered.

This study incorporates two mitochondrial (COI and 16S) and two nuclear loci (two regions of 28S (D1–3 and D7–10) and histone‐3). COI and 16S are particularly useful for evaluating population‐level variation as they exhibit high levels of genetic variation and have been used extensively for species‐ and population‐level phylogenetic research in springtails (Hogg & Hebert, 2004). Collembola are generally characterized by extremely high levels of molecular diversity (Katz et al., 2015); therefore, more slowly evolving loci, 28S and histone‐3, were included to provide stronger phylogenetic signal among more distantly related taxa. Histone‐3 and 28S D1–3 were excluded for *Pogonognathellus* due to inconsistent amplification. See Supporting information Appendix S1 for list of all taxa included in this study, including sample information and all sequences with corresponding GenBank (Benson et al., 2013) accession numbers. See Supporting information Appendix S2 for PCR and sequencing primers, including a description of the PCR protocol and sequence alignment methods used in this study. The outgroup taxa listed in Supporting information Appendix S3 were chosen based on their affinities with the target taxa and availability of sequences in GenBank.

### 2.3 Detecting and delimiting cryptic diversity

The presence of cryptic diversity was detected by incorporating a number of different tests. First, we calculated uncorrected pairwise COI distance frequencies for all sampled specimens with PAUP* 4.0a build 159 (Swofford, 2002) and plotted distance frequency histograms to detect the presence of interspecific variation within each targeted morphospecies. A gap between the greatest putative intraspecific and smallest putative interspecific pairwise distances can be interpreted as the boundary between species‐ and population‐level variation (Meier, Zhang, & Ali, 2008).

To determine how interspecific variation was geographically distributed, we performed a hierarchical analysis of molecular variance (AMOVA) for COI, 16S, and 28S using all taxa sampled for each target morphospecies using Arlequin v. 3.5.2.2 (Excoffier & Lischer, 2010). Haplotypes were grouped within samples, among samples in caves, and among caves with 50,000 permutations performed to assess significance. The presence of strong genetic structuring within samples or among samples in caves can be an indicator of cryptic diversity because sexual isolation is typically required to maintain high levels of genetic variation occurring in sympatry.

We also delimited putative species boundaries using a General Mixed Yule Coalescent (GMYC) analysis (Pons et al., 2006). This method uses ultrametric gene trees to identify the interface between population‐ and species‐level branching patterns and demarcates genetically cohesive clades as independent evolutionary units known as operational taxonomic units (OTUs). The GMYC analysis was performed on COI gene trees using the single threshold delimitation method implemented in the splits package (Ezard, Fujisawa, & Barraclough, 2009) in R (R Core Team, 2017). Bayesian inference of COI gene trees used for the GMYC analysis was conducted independently for both genera and performed using BEAST2 v. 2.4.8 (Bouckaert et al., 2014) with the following parameters: Site model averaging implemented in bModelTest (Bouckaert & Drummond, 2017) was used to accommodate uncertainty in the model of sequence evolution (default parameters); a strict clock rate set to 1 for relative branch length estimation; Yule tree model; monophyletic constraint prior on the ingroup taxa; Markov chain Monte Carlo (MCMC) for 100 million generations; and sampling statistics and trees every 1,000 generations (10% burn‐in). Effective sample size (ESS) for all parameters was determined to be greater than 200 with Tracer v1.6 (Rambaut, Suchard, Xie, & Drummond, 2014). Maximum clade credibility trees were inferred with TreeAnnotator v2.4.8 (Bouckaert et al., 2014). bModelTest site model distributions and statistics are reported in Supporting information Appendices S4a and S5a.

Inter‐ and intra‐OTU uncorrected genetic distances for all loci were computed in PAUP* and plotted in R. Representative specimen vouchers recovered during DNA extraction for each OTU were directly slide‐mounted with Hoyer's medium (Mari‐Mutt, 1979) for morphological examination using a Nikon Eclipse Ni‐U upright microscope with phase contrast to check for morphological differentiation among OTUs, the presence of troglomorphy, and to provide preliminary species identifications for focal OTUs.

### 2.4 Tests for genetic structure

The relative role of cave dependence and its influence on springtail dispersal capacity remain unclear, in part, because the identities of genetic barriers are not known for cave‐dwelling springtails. To identify barriers to *Pygmarrhopalites* and *Pogonognathellus* dispersal, we evaluated and compared levels of genetic structure across cave boundaries and the Mississippi River Valley. In addition, we also included sinkhole area boundaries in the genetic structure analyses. Because cave density in karst regions can be correlated with sinkhole density (Shofner, Mills, & Duke, 2001), areas without sinkholes may lack sufficient cave habitat for subterranean species dispersal. Therefore, we assigned discontinuous sinkhole karst areas in Illinois (Panno, Weibel, Wicks, & Vandike, 1999; Panno et al., 1997; Venarsky, Anderson, & Wilhelm, 2009) and Missouri (Burr, Adams, Krejca, Paul, & Warren, 2001; Panno et al., 1999) (neighboring karst subregions were combined) to each cave for genetic structure analyses (Table 1; Figure 1).

The most sampled OTUs for each target morphospecies, identified by the GMYC analysis, were chosen as focal OTUs for population analyses to avoid attributing deeply divergent and structured lineages to population‐level variation, rather than to species‐level variation (Fouquet et al., 2007). Hierarchical AMOVAs were performed independently with Arlequin for COI and 16S for both focal OTUs by grouping haplotypes within samples, among samples within barriers, and among samples across barriers. Significance was assessed with 50,000 permutations.

Patterns of population structure resulting from dispersal and genetic drift, rather than of vicariance across geographic barriers, are common in animals with low mobility and can usually be attributed to a model of IBD (Costa et al., 2013; Timmermans et al., 2005). To determine whether geographic distance is significantly correlated with genetic distance, we performed a Mantel test (Mantel, 1967; Sokal, 1979; but see Diniz‐Filho et al., 2013; Legendre, Fortin, & Borcard, 2015) for each locus. We also evaluated the significance of genetic structure across barriers while controlling for geographic distance using a partial Mantel test (Smouse, Long, & Sokal, 1986), which allows for the comparison of two variables (i.e., pairwise genetic distances and position relative to geographic barrier) while controlling a third (i.e., geographic distances). The partial Mantel tests required matrices of pairwise uncorrected genetic distances for both focal OTUs, geographic distances (great‐circle distance) between each cave location, and matrices with variables coded to indicate whether each pair of specimens occurred together or on different sides of each geographic barrier. All simple and partial Mantel tests were calculated with zt v1.1 (Bonnet & Van de Peer, 2002) with 100,000 permutations.

Templeton‐Crandall‐Sing (TCS) haplotype networks (Clement, Snell, & Walker, 2002) for COI and 16S were estimated with PopART (Leigh & Bryant, 2015) to visualize and compare phylogeographic structure across genetic barriers for *Pygmarrhopalites* and *Pogonognathellus* focal OTUs.

### 2.5 Phylogenetic inference, divergence time estimation, and topology tests

To further investigate the interplay of vicariance and dispersal capacity on cave springtail diversity, we conducted a Bayesian phylogenetic analysis using BEAST 2 to infer evolutionary relationships and to estimate divergence times for all sampled lineages of *Pygmarrhopalites* and *Pogonognathellus*. Two independent datasets were analyzed and compared: the *Pygmarrhopalites* dataset (COI, 16S, 28S D1–3, 28S D7–10, histone‐3; 3,358 total bp) and the *Pogonognathellus* dataset (COI, 16S, 28S D7–10; 2,059 total bp). External rates were used for molecular clock calibrations rather than fossil information because springtails lack an adequate fossil record and phylogenetic framework for calibrating molecular clocks. Katz (2018) expanded upon Cicconardi, Nardi, Emerson, Frati, and Fanciulli's (2010) springtail rate assessment by evaluating relative substitution rates for COI (rather than COII) across the Hexapoda and found that COI rates are similar among most hexapod groups (including Collembola), suggesting that the use of “universal” COI insect clocks is likely appropriate for estimating springtail divergence times. Brower's (1994) estimate of 2.3% divergence per million years for COI is the most widely used external rate calibration for inferring arthropod divergence times. However, this rate has been recently criticized for poor statistical rigor (Papadopoulou, Anastasiou, & Vogler, 2010); therefore, we decided to use the more statistically robust rates estimated by Papadopoulou et al. (2010) for molecular clock calibration: COI = 3.54%/Ma, 16S = 1.08%/Ma, 28S = 0.12%/Ma. Clock rate parameters in BEAST 2 were set to 0.0168, 0.0054, and 0.0006 substitutions/site/Ma, respectively. Clock and site models for each partition were unlinked, and tree models were linked for all gene partitions. For the *Pygmarrhopalites* dataset, 28S regions D1–3 and D7–10 were combined into a single 28S clock model partition, but site models were estimated independently with bModelTest for each region. Rates for histone‐3 (*Pygmarrhopalites* only) were estimated. Relaxed log‐normal clock models were applied to all gene partitions. Analyses were run for 200 million generations (five independent runs), sampling statistics, and trees every 5,000 generations. All additional parameters were the same as those used to estimate COI gene trees for the GMYC analysis (see above). Convergence (ESS > 200) and burn‐in (10%) were assessed with Tracer, and maximum clade credibility trees were inferred with TreeAnnotator. bModelTest site model distributions and statistics are reported in Supporting information Appendices S4b–c and S5b–c.

We also compared different topological models using Bayes factors (BF) to further test for reciprocal monophyly across the Mississippi River for *Pygmarrhopalites* and *Pogonognathellus* focal OTUs. Informed topology was strictly constrained in the prior for all hypotheses because irrelevant background signal in an unconstrained analysis can bias BF tests for monophyly (Bergsten, Nilsson, & Ronquist, 2013). For the *Pygmarrhopalites* dataset, we compared three different hypotheses regarding the uncertain placement of focal OTU Illinois lineages from Indian Cave (INC) (see results): *H*_0_, all focal OTU Illinois lineages (including INC) were constrained to be monophyletic and sister to all focal OTU Missouri lineages [(IL + INC) + MO]; *H*_1_, all focal OTU Missouri lineages and focal OTU lineages from INC were constrained as monophyletic and sister to all other focal OTU Illinois lineages [(MO + INC) + IL]; and *H*_2_, focal OTU INC lineages were constrained to be sister to all other focal OTU lineages [INC + (IL + MO)]. Two models were compared for the *Pogonognathellus* dataset: *H*_0_, focal OTU Illinois and Missouri lineages were each constrained as monophyletic and sister to each other [IL + MO]; *H*_1_, relationships that group focal OTU Missouri and Illinois lineages resulting from the unconstrained analysis (best tree) were constrained [TMC + (ILC + HSC) & (AC1 + AC2) + (PAC + FPC+MJP)] (see Table 1 for cave abbreviations). Marginal log likelihoods were estimated with stepping‐stone MCMC sampling using the MODEL_SELECTION v. 1.3.4 package in BEAST2 (alpha = 0.3; steps = 100; chain length = 2 million; all other parameters as default). Number of steps and chain length were increased until there was no significant difference in marginal likelihood estimates. Excluding topological constraint priors, all other parameters were identical for each model; same as those used for divergence time estimations (see above). Following guidelines proposed by Kass and Raftery (1995), a twice logarithm BF difference (2 × log_e_BF) of higher than 6 was considered strong evidence against the null hypothesis.

## 3 RESULTS

### 3.1 Evidence for cryptic diversity

**Figure 3:**
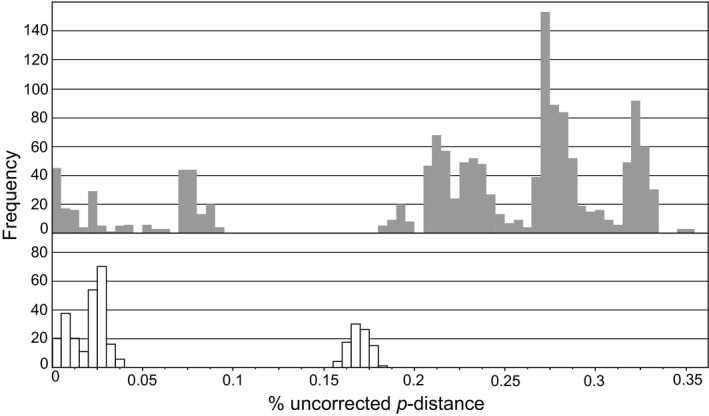
COI pairwise distance (uncorrected) frequency histogram for *Pygmarrhopalites* (gray) and *Pogonognathellus* (white)

Uncorrected pairwise COI distance frequency histograms revealed extraordinarily high genetic distances among sampled specimens within each morphospecies: up to 35% for *Pygmarrhopalites* and 18% for *Pogonognathellus* (Figure 3). COI distances above 8%–15% in springtails are typically recognized as interspecific when used in combination with independent evidence (Katz et al., 2015). Moreover, COI distances form bimodal distributions for both morphospecies, each separated by a 10% gap (Figure 3) which can be interpreted as a boundary between intra‐ and interspecific genetic variation (Meier et al., 2008), providing preliminary support for the presence of cryptic diversity within both target morphospecies.

The results of the initial AMOVA that incorporated all sampled taxa identified high levels of genetic structure within caves and within samples, supporting the presence of sympatric cryptic species (Table 2): Between 40% and 60% of genetic variation in COI, 16S, and 28S was structured among samples within the same cave for both genera. Genetic variation in COI, 16S, and 28S (24%, 21%, and 29%, respectively) was also structured within samples for *Pygmarrhopalites*, but this pattern was not recovered for *Pogonognathellus* (COI, 2%; 16S, 0%; 28S, 41%).

**Table 2:** Hierarchical analysis of molecular variance (AMOVA) of COI, 16S, and 28S for all sampled lineages of *Pygmarrhopalites* and *Pogonognathellus* grouped by cave

Source of variation	COI					16S					28S				
	*df*	SS	VC	V%	*ϕ*‐Statistics	*df*	SS	VC	V%	*ϕ*‐Statistics	*df*	SS	VC	V%	*ϕ*‐Statistics
*Pygmarrhopalites*															
Among caves	15	1,785.70	20.53	28.62	*ϕ*ct = 0.29**	14	658.50	6.60	22.48	*ϕ*ct = 0.23*	15	346.28	1.80	10.70	*ϕ*ct = 0.11
Among samples within caves	13	829.91	34.20	47.68	*ϕ*sc = 0.67**	12	343.73	16.46	56.08	*ϕ*sc = 0.72**	13	238.20	10.00	60.00	*ϕ*sc = 0.67**
Within samples	13	221.08	17.01	23.71	*ϕ*st = 0.76***	12	75.50	6.29	21.44	*ϕ*st = 0.79***	11	53.80	4.90	29.30	*ϕ*st = 0.71***
*Pogonognathellus*															
Among caves	10	337.90	11.61	56.37	*ϕ*ct = 0.56*	9	193.08	7.66	60.61	*ϕ*ct = 0.61*	9	11.27	0.03	2.50	*ϕ*ct = 0.03
Among samples within caves	13	130.02	8.49	41.21	*ϕ*sc = 0.94	12	67.50	4.98	39.39	*ϕ*sc = 1.00	11	13.50	0.68	56.22	*ϕ*sc = 0.58
Within samples	2	1.00	0.50	2.43	*ϕ*st = 0.98*	2	0.00	0.00	0.00	*ϕ*st = 1.00*	1	0.50	0.50	41.28	*ϕ*st = 0.03

*Notes*

*df*: degrees of freedom; SS: sum of squares; VC: variance components; V%: percent of variation.

Significance is based on 50,000 permutations: *p < 0.05, **p < 0.01, ***p < 0.001.

**Figure 4:**
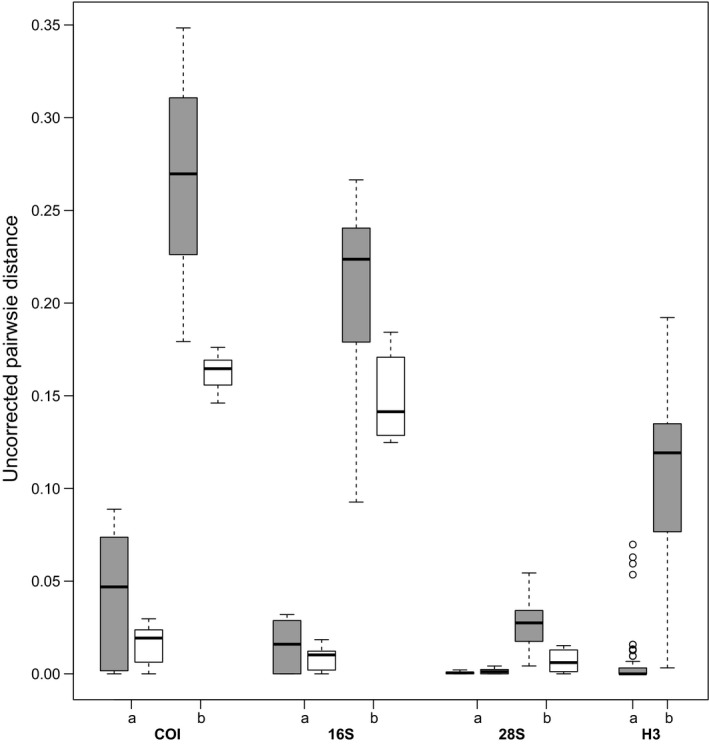
Boxplots comparing (a) inter‐ and (b) intra‐OTU genetic distances (uncorrected) for *Pygmarrhopalites* (gray, left) and *Pogonognathellus* (white, right)

The GMYC analyses revealed 14 putative species: 10 *Pygmarrhopalites* OTUs (A1–10) and four *Pogonognathellus* OTUs (T1–4) (Figure 6). Intra‐OTU % distances for COI ranged from 18% to 35% (mean = 27%) for *Pygmarrhopalites* and 15% to 18% (mean = 16%) for *Pogonognathellus*, while intra‐OTU % distances for COI ranged from 0% to 9% (mean = 4%) for *Pygmarrhopalites* and 0% to 3% (mean = 2%) for *Pogonognathellus*. Although *Pygmarrhopalites* had more variable and notably higher genetic distances compared to *Pogonognathellus*, there is no overlap between intra‐ and inter‐OTU COI and 16S distances for either genus and mean inter‐OTU distances are substantially higher than intra‐OTU distances for 28S and H3 (Table 3; Figure 4). *Pygmarrhopalites* A10 and *Pogonognathellus* T4 were chosen for comparative phylogeographic analysis because they included the largest number of sampled lineages. It is worth noting that because OTUs could not be differentiated under stereomicroscopy, targeted sequencing to increase a specific OTU's sample size was not possible.

**Table 3:** Summary statistics of intra‐ and inter‐OTU genetic distances (uncorrected) for each locus evaluated for both focal morphospecies

	*n*	Mean	*SD*	*SE*	Range
*Pygmarrhopalites*					
COI Intra‐OTU	130	0.0397	0.0332	0.0029	0.0000–0.0888
COI Inter‐OTU	731	0.2659	0.0421	0.0016	0.1792–0.3484
16S Intra‐OTU	118	0.0141	0.0122	0.0011	0.0000–0.0320
16S Inter‐OTU	623	0.2099	0.0376	0.0083	0.0927–0.2664
28S Intra‐OTU	130	0.0005	0.0006	0.0001	0.0000–0.0021
28S Inter‐OTU	603	0.0260	0.0108	0.0004	0.0079–0.0544
H3 Intra‐OTU	130	0.0038	0.0109	0.0010	0.0000–0.0698
H3 Inter‐OTU	731	0.1084	0.0371	0.0014	0.0032–0.1922
*Pogonognathellus*					
COI Intra‐OTU	232	0.0157	0.0095	0.0006	0.0000–0.0297
COI Inter‐OTU	93	0.1624	0.0072	0.0007	0.1461–0.1761
16S Intra‐OTU	210	0.0083	0.0058	0.0004	0.0000–0.0184
16S Inter‐OTU	66	0.1474	0.0188	0.0023	0.1248–0.1843
28S Intra‐OTU	190	0.0014	0.0012	0.0001	0.0000–0.0042
28S Inter‐OTU	63	0.0068	0.0052	0.0007	0.0000–0.0152

*Note*

*SD*: standard deviation; *SE*: standard error.

Morphological examination of slide‐mounted DNA voucher specimens of *Pygmarrhopalites* revealed similar, but distinct and unique morphologies for all OTUs (e.g., differentiation of the female subanal appendage and claw morphology) that do not fit any known species description. *Pygmarrhopalites* A10 (Figure 2a) had moderate troglomorphy (e.g., elongated antennae and thread‐like unguiculus) and was most similar to *Pygmarrhopalites pavo* (Christiansen & Bellinger, 1996), a troglobiont reported from caves in Virginia (Christiansen & Bellinger, 1996), West Virginia (Fong, Culver, Hobbs, & Pipan, 2007), Tennessee (Lewis, 2005), and Missouri (Zeppelini, Taylor, & Slay, 2009). We believe the unique differences in morphology, in combination with molecular evidence, support the recognition of all *Pygmarrhopalites* OTUs as distinct and potentially new species. Because some cryptic lineages may be of higher conservation concern, it is imperative to identify and describe these lineages for potential management initiatives (Delić, Trontelj, Rendoš, & Fišer, 2017; Niemiller, Graening, et al., 2013). However, we chose to refrain from giving OTUs formal species names at this time because a comprehensive taxonomic review is required to describe new species and to clarify the status of existing species—a task beyond the scope of this study.

All sampled lineages of *Pogonognathellus* were identified as members of the *Pogonognathellus* pale species complex (Felderhoff et al., 2010), (Figure 2b), a common eutroglophile inferred to be comprised of multiple cryptic species that cannot be differentiated without molecular data (Felderhoff et al., 2010), a finding that is also supported here by the recovery of four deeply divergent molecular lineages (T1–T4) with indistinguishable morphology under compound light microscopy.

### 3.2 Genetic structure

Results of the hierarchical AMOVAs identified that the majority of genetic variation in COI and 16S was structured among caves for both *Pygmarrhopalites* A10 (COI, 88%; 16S, 91%) and *Pogonognathellus* T4 (COI, 92%; 16S, 98%) (Table 4a). Genetic variation in COI and 16S was also strongly structured among sinkhole areas (COI, 94%; 16S, 96%) and regions east and west of the Mississippi River (COI, 58%; 16S, 73%) for *Pygmarrhopalites* A10, contrasting sharply with patterns of genetic variation observed for *Pogonognathellus* T4 populations spanning sinkhole area boundaries (COI, 43%; 16S, 51%) and regions across the Mississippi River (COI, 7%; 16S, 7%) (Table 4b–c).

**Table 4:** Hierarchical analysis of molecular variance (AMOVA) of COI and 16S for *Pygmarrhopalites* and *Pogonognathellus* focal OTUs grouped by (a) cave, (b) sinkhole area, and (c) by region relative to the Mississippi River

Source of variation	COI					16S				
	*df*	SS	VC	V%	*ϕ*‐Statistics	*df*	SS	VC	V%	*ϕ*‐Statistics
(a) Cave										
*Pygmarrhopalites* A10										
Among caves	4	170.58	15.68	87.98	*ϕ*ct = 0.88	4	31.25	2.93	90.72	*ϕ*ct = 0.91
Among pops within caves	2	5.25	1.93	10.85	*ϕ*sc = 0.9	2	0.75	0.3	9.28	*ϕ*sc = 1
Within populations	6	1.25	0.21	1.17	*ϕ*st = 0.99***	6	0	0	0	*ϕ*st = 1***
*Pogonognathellus* T4										
Among caves	8	104.05	5.87	92.47	*ϕ*ct = 0.93***	7	40	2.49	97.91	*ϕ*ct = 0.98***
Among pops within caves	11	5.22	−0.02	−0.35	*ϕ*sc = −0.05	11	0.67	0.53	2.09	*ϕ*sc = 1
Within populations	2	1	0.5	7.88	*ϕ*st = 0.92*	2	0	0	0	*ϕ*st = 1*
(b) Sinkhole area										
*Pygmarrhopalites* A10										
Among sinkhole areas	3	170.16	19.08	94.16	*ϕ*ct = 0.95*	3	31.25	3.54	96.06	*ϕ*ct = 0.96**
Among pops within areas	3	5.67	0.98	4.82	*ϕ*sc = 0.82	3	0.75	0.15	3.94	*ϕ*sc = 1
Within populations	6	1.25	0.21	1.03	*ϕ*st = 0.99***	6	0	0	0	*ϕ*st = 1***
*Pogonognathellus* T4										
Among sinkhole areas	2	36.67	3	43.33	*ϕ*ct = 0.43***	2	16.27	1.42	50.83	*ϕ*ct = 0.51***
Among pops within areas	17	72.6	3.42	49.44	*ϕ*sc = 0.87	16	24.4	1.38	49.17	*ϕ*sc = 1
Within populations	2	1	0.5	7.23	*ϕ*st = 0.93*	2	0	0	0	*ϕ*st = 1
(c) Region										
*Pygmarrhopalites* A10										
Among regions	1	99.38	13.59	58.36	*ϕ*ct = 0.58*	1	21.92	3.38	72.7	*ϕ*ct = 0.73*
Among pops within regions	5	76.44	9.49	40.75	*ϕ*sc = 0.98**	5	10.83	1.27	27.3	*ϕ*sc = 1*
Within populations	6	1.25	0.2	0.89	*ϕ*st = 0.99***	6	0	0	0	*ϕ*st = 1***
*Pogonognathellus* T4										
Among regions	1	7.29	0.38	6.77	*ϕ*ct = 0.07	1	2.83	0.15	6.79	*ϕ*ct =0.07
Among pops within regions	18	101.98	4.7	84.26	*ϕ*sc = 0.9	17	37.83	2.01	93.21	*ϕ*sc = 1
Within populations	2	1	0.5	8.97	*ϕ*st = 0.91*	2	0	0	0	*ϕ*st = 1

*Notes*

*df*: degrees of freedom; SS: sum of squares; VC: variance components; V%: percent of variation.

Significance is based on 50,000 permutations: *p < 0.05, **p < 0.01, ***p < 0.001.

The Mantel test recovered significant IBD patterns for both focal OTUs, although the relationship between genetic distance and geographic distance was more strongly correlated for *Pygmarrhopalites* A10 (COI, *r* = 0.79; 16S, *r* = 0.79) compared to *Pogonognathellus* T4 (COI, *r* = 0.37; 16S, *r* = 0.45) (Table 5). For *Pygmarrhopalites* A10, genetic distance remained strongly correlated to sinkhole area (COI, *r* = 0.63; 16S, *r* = 0.5) and position relative to the Mississippi River (COI, *r* = 0.54; 16S, *r* = 0.85) after controlling for geographic distance using partial Mantel tests, while *Pogonognathellus* T4 had weakly positive to slightly negative correlations between genetic distance and sinkhole area (COI, *r* = −0.19; 16S, *r* = 0.28) and position relative to the Mississippi River (COI, *r* = −0.21; 16S, *r* = −0.23). However, the strong patterns of genetic structure among caves for *Pygmarrhopalites* A10 recovered by the AMOVA were weakly or not supported after controlling for geographic distance with partial Mantel tests (COI, *r* = 0.27; 16S, *r* = 0.18). In contrast, strong positive correlations between genetic distance and caves were detected for *Pogonognathellus* T4 (COI, *r* = 0.62; 16S, *r* = 0.63; Table 5).

**Table 5:** Mantel test results (a, COI; b, 16S) to identify isolation‐by‐distance (IBD) patterns and correlations between genetic distance and geographic barriers after controlling for geographic distance in *Pygmarrhopalites* A10 and *Pogonognathellus* T4

Barrier	*R* value	
	*Pygmarrhopalites* A10	*Pogonognathellus* T4
(a)		
Geographic distance	**0.79** ***	0.37**
Cave boundaries	0.27*	0.62****
Sinkhole area boundaries	**0.63** ***	−0.19*
Mississippi River Valley	**0.54** ***	−0.21*
(b)		
Geographic distance	**0.79** ***	0.45**
Cave boundaries	0.18	**0.63** ***
Sinkhole area boundaries	**0.5** ***	0.28**
Mississippi River Valley	**0.85** ***	−0.23**

*Notes*

Bold values indicate strongly positive correlations.

Significance is based on 100,000 permutations: *p < 0.05, **p < 0.01, ***p < 0.001.

**Figure 5:**
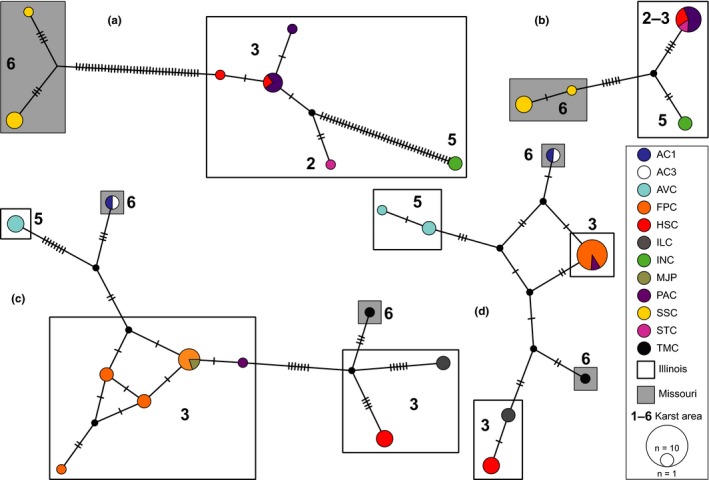
TCS haplotype networks of *Pygmarrhopalites* A10 (a, COI; b, 16S) and *Pogonognathellus* T4 (c, COI; d, 16S). Hatch marks represent mutational steps between haplotypes; circle color indicates cave locality as illustrated in the legend; numbers 1–6 represent sinkhole karst area (see Table 1; Figure 1); rectangle color indicates position relative to the Mississippi River (white, Illinois; gray, Missouri); node diameter represents sample size

Templeton‐Crandall‐Sing haplotype networks for COI and 16S revealed concordant relationships, but also showed markedly distinct phylogeographic patterns between *Pygmarrhopalites* A10 and *Pogonognathellus* T4 (Figure 5). *Pygmarrhopalites* A10 haplotypes were geographically structured with all haplotypes segregating by position relative to the Mississippi River and sinkhole area (although 16S haplotypes were shared among neighboring sinkhole areas 2 and 3), whereas *Pogonognathellus* T4 haplotypes did not strongly segregate by geographic barrier, with divergent haplotypes occurring together within sinkhole areas and on both sides of the Mississippi River Valley. Additionally, the *Pygmarrhopalites* A10 COI haplotype network clearly illustrates significant levels of sequence divergence between populations spanning the Mississippi Valley (mean = 7.5%) and between populations from INC and other Illinois caves (mean = 7.4%; Figure 5).

### 3.3 Phylogeny, divergence times, and topology tests

**Figure 6:**
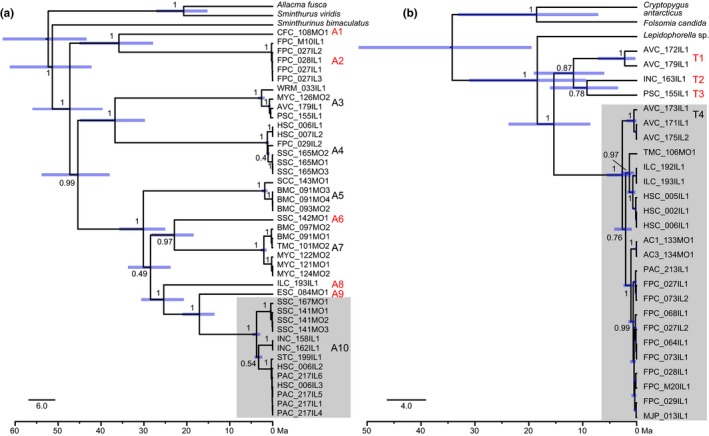
Time‐calibrated trees for (a) *Pygmarrhopalites* and (b) *Pogonognathellus* inferred by Bayesian phylogenetic analysis. Clade posterior probabilities are indicated at each node. Divergence times are represented by blue bars at each node with their length corresponding to the 95% HPD of node ages. OTUs identified by the GMYC analysis are indicated to the right of each clade (A1–A10 and T1–T4). Focal OTUs chosen for population structure analyses (A10 and T4) are highlighted in gray boxes (see Figure 7 for close‐up of A10). Single‐site endemic OTUs are labeled in red. Taxon labels correspond to cave name abbreviation, sample #, state, specimen # (see Table 1 for cave abbreviations and Appendix 1 for sample information). Scale bars represent substitutions/site/Ma

The rate‐calibrated phylogenetic analysis based on the multilocus dataset produced trees with high support for all OTUs identified by the GMYC analysis, and molecular divergence time estimates revealed that all OTU diversification predated the Pliocene (Figure 6). The median age of the most recent common ancestor (MRCA) for all sampled lineages of *Pygmarrhopalites* was estimated to be 47.34 Ma (95% highest posterior density [95% HPD] = 39.62–55.98 Ma), and the MRCA of all sampled lineages of *Pogonognathellus* is 15.36 Ma (95% HPD = 8.59–23.77 Ma). The median age of *Pygmarrhopalites* A10 MRCA is 3.77 Ma (95% HPD = 2.90–4.76 Ma), and *Pogonognathellus* T4 MRCA is 2.67 (95% HPD = 1.2–5.6 Ma). Reciprocal monophyly across the Mississippi River was only recovered for *Pygmarrhopalites* A10, albeit with low clade support grouping lineages from INC with other Illinois lineages from Stemmler Cave (STC), Hoppy Speck Cave (HSC), and Pautler Cave (PAC). *Pygmarrhopalites* A10 populations from INC diverged from other Illinois lineages ~3.34 Ma (95% HPD = 2.43–4.24 Ma), similar to divergence times estimated for Illinois and Missouri populations spanning the Mississippi River ~3.77 Ma (95% HPD = 2.90–4.76 Ma). There was no evidence of vicariance for *Pogonognathellus* T4 lineages, as they were not grouped by position relative to the Mississippi River.

The multilocus phylogeny also shows that two additional OTUs (*Pygmarrhopalites* A3 and A4) contain both Illinois and Missouri lineages, but did not form monophyletic groups by region relative to the Mississippi River. All other OTUs were short‐range endemics, from a single cave (A1, A2, A6, A8, A9, T1–3) or from neighboring cave systems within the same sinkhole area (A5, A7) (Figure 6). Subsequent independent runs (*n* = 4) produced congruent topological relationships and divergence times.

The topology tests (Table 6) strongly supported a hypothesis of reciprocal monophyly across the Mississippi River (*H*_0_) for *Pygmarrhopalites* A10 over alternative topological hypotheses *H*_1_ (2 × log_e_BF = 19.94) and *H*_2_ (2 × log_e_BF = 11.46). However, reciprocal monophyly (*H*_0_) was strongly rejected in favor of topology *H*_1_ for *Pogonognathellus* T4 (2 × log_e_BF = −104.82), supporting more recent gene flow across the Mississippi River Valley.

**Table 6:** Bayes factor comparisons of marginal likelihood estimates from stepping‐stone sampling analysis for each topological hypothesis (*H*_0_– *H*_2_) to determine support for reciprocal monophyly across the Mississippi Valley (*H*_0_) for *Pygmarrhopalites* A10 and *Pogonognathellus* T4

	*H*_0_	*H*_1_	*H*_2_	2 × log_e_ BF *H*_0_ vs. *H*_1_	2 × log_e_ BF *H*_0_ vs. *H*_2_
*Pygmarrhopalites* A10	−16,060.28	−16,070.25	−16,066.01	**19.94**	**11.46**
*Pogonognathellus* T4	−7,349.78	−7,297.37	—	−104.82	—

*Notes*

BF values in bold indicate strong support for *H*_0_.

*H*_0_ = IL + MO.

*H*_1_ (*Pygmarrhopalites*) = (INC + SSC) + (STC + HSC + PAC); *H*_1_ (*Pogonognathellus*) = TMC + (ILC + HSC) & (AC1 + AC2) + (PAC + FPC + MJP).

*H*_2_ = INC + ((SCC) + (STC + HSC + PAC)).

## 4 DISCUSSION

### 4.1 Comparative phylogeography

**Figure 7:**
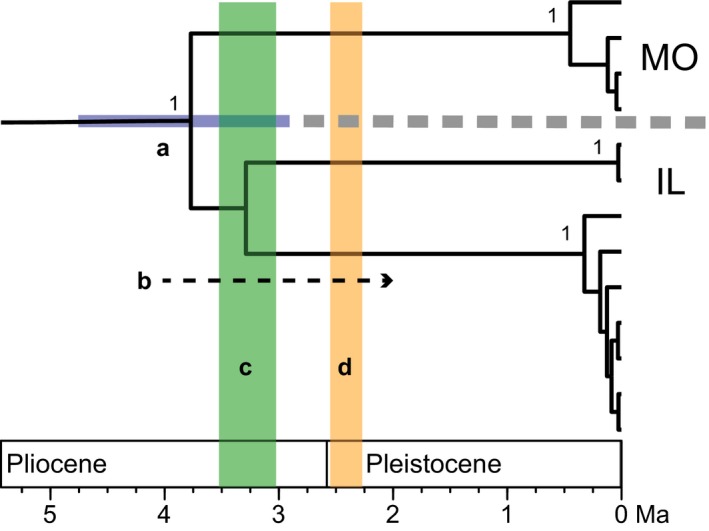
Close‐up of clade *Pygmarrhopalites* A10 from Figure 6a illustrating timing information from estimates of molecular divergence and geological evidence supporting vicariance across the Mississippi River Valley: (a) 2.90–4.76 Ma (95% HDP) (blue bar) divergence time between Missouri and Illinois lineages (separated by gray dashed line), posterior probabilities at each node lower than 1 are not displayed; (b) late Pliocene/early Pleistocene timing (dashed arrow) of initial Mississippi River entrenchment (Cupples & Van Arsdale, 2014) and increased river discharge (Cox et al., 2014); (c) 3.25 ± 0.26 Ma (green column) timing of initial Green River karst incision and excavation (Granger et al., 2001); (d) 2.41 ± 0.14 Ma (orange column) timing of first glacial melt (Balco et al., 2005). Holocene (0.01 Ma to present) is not labeled

We found that the troglobiont, *Pygmarrhopalites* A10, and the eutroglophile, *Pogonognathellus* T4, have different phylogeographic patterns despite being codistributed across the same geological landscape. Population structure analyses (Tables 4, 5; Figure 5), time‐calibrated phylogenetic reconstructions (Figure 6), and topology tests (Table 6) indicated that intrinsic differences between these species, such as their degree of cave dependence, have had major impacts on processes involved in promoting and maintaining genetic isolation in this system (i.e., vicariance and dispersal). Specifically, two important patterns emerged from the comparative phylogeographic analysis. First, sinkhole area boundaries and the Mississippi River Valley were identified as significant dispersal barriers for *Pygmarrhopalites* A10 only. Hierarchical AMOVAs initially revealed that more than half of all genetic variation was distributed among caves, sinkhole areas, and across the Mississippi River Valley for *Pygmarrhopalites* A10, but for *Pogonognathellus* T4, comparable levels of genetic structure were recovered only among caves (Table 4). Mantel tests confirmed geographic distance to be a significant driver of genetic isolation for both taxa (Table 5), suggesting springtails are weak dispersers regardless of ecological classification. After controlling for geographic distance using partial Mantel tests, we still recovered significant positive correlations between genetic distance and sinkhole areas and between genetic distance and position relative to the Mississippi River for *Pygmarrhopalites*, but not for *Pogonognathellus* (Table 5). The haplotype networks (Figure 5), phylogenetic trees (Figures 6 and 7), and topology tests (Table 6) also corroborate these findings providing similar patterns of genetic structure across sinkhole area boundaries and the Mississippi River Valley. These results indicate that isolation maintained by the Mississippi River valley floodplain and sinkhole area boundaries are driving genetic differentiation in troglobiotic *Pygmarrhopalites*, while IBD is the primary driver of genetic differentiation in eutroglophilic *Pogonognathellus*.

Relative to the Mississippi River Valley and sinkhole area boundaries, we observed a very different pattern when genetic variation was partitioned among caves: Cave boundaries were identified as significant genetic barriers for *Pogonognathellus* only, whereas patterns of genetic structure among caves identified by the AMOVA for *Pygmarrhopalites* A10 (Table 4a) were not supported after accounting for geographic distance (Table 5). In this case, patterns of genetic structure among caves are driven by IBD for *Pygmarrhopalites* A10 (not *Pogonognathellus* T4) suggesting that troglobiotic *Pygmarrhopalites* are capable of dispersing between caves. Although this finding appears to contradict the hypothesis that troglobiotic species are less capable of dispersal across geographic barriers, it can still be explained by differences in cave habitat preferences. Aquatic interstitial subterranean connections joining neighboring cave systems may enable subterranean dispersal during flooding events for *Pygmarrhopalites* A10. This is supported by shared 16S haplotypes between neighboring cave systems (PAC, HSC, and STC) (Figure 5b). Groundwater connections (e.g., alluvial aquifers, epikarst systems) have been implicated as “interstitial highways” that can provide subsurface dispersal pathways for a wide range of subterranean arthropods (e.g., Lefébure et al., 2006; Ward & Palmer, 1994), but Collembola are not normally considered members of the interstitial groundwater community as they cannot complete life cycles while submerged (Deharveng, D'Haese, & Bedos, 2008). However, growing evidence suggests that they are not only present in these habitats, but can occur in abundance and comprise diverse communities (Bretschko & Christian, 1989; Deharveng et al., 2008; Palacios‐Vargas, Cortés‐Guzmán, & Alcocer, 2018; Shaw, Dunscombe, & Robertson, 2011). Shaw et al. (2011) documented a unique springtail community from a karstic hyporheic zone that includes species previously associated only with caves. Under this scenario, obligate associations with certain cave habitats may actually facilitate dispersal, colonization, and gene flow among caves connected by subterranean passages.

Cave‐to‐cave subterranean dispersal is unlikely or infrequent for *Pogonognathellus* because species in this genus do not occur in interstitial habitats and prefer floor or wall surfaces near cave entrances rather than dark zone habitats. This is supported by strong genetic structuring among caves for *Pogonognathellus* T4 indicating that cave‐to‐cave dispersal is extremely rare for this species despite having naturally occurring surface populations that could presumably facilitate gene flow between caves. Cave‐to‐cave surface dispersal may be also difficult for this species simply because cave entrances are extremely small features within very large landscapes (Culver & Pipan, 2009b). Long‐term local persistence of cave populations coupled with long‐distance dispersal and gene flow contributed by surface populations may explain the presence of both isolation‐by‐distance patterns across sinkhole area boundaries and river barriers and strong isolation‐by‐cave patterns in *Pogonognathellus*. However, additional sampling of surface populations for genetic analysis is necessary for a better understanding of the mechanisms driving patterns of genetic structure in *Pogonognathellus* cave populations.

To assess the effect of cave dependence on patterns of molecular variation, we were required to make informed assumptions about species ecology, including the classification of *Pygmarrhopalites* A10 as a troglobiont. For many small cave‐dwelling animals, such as springtails, it is often impossible to ascertain with certainty that a species only occurs in caves (Christiansen, 1962); a species reported only from caves could also be a common soil species, having yet to be reported from surface habitats; the distinction between cavernicolous habitats and other subsurface microhabitats may be weak or nonexistent for small animals; and troglobionts often lack obvious troglomorphy. Despite these concerns, we are confident that the combination of troglomorphy, close morphological affinities to known troglobiotic species, and their exclusive occurrence in dark or deep twilight cave zones (Supporting information Appendix S1) provides sufficient evidence that *Pygmarrhopalites* A10 is a troglobiont.

The degree of cave dependence is certainly a major factor influencing dispersal capacity in cave‐dwelling organisms, but additional intrinsic differences between *Pygmarrhopalites* and *Pogonognathellus* may have also contributed to the disparate phylogeographic patterns observed in this study. The genera being compared belong to separate orders of Collembola, differing substantially in size, mobility, and life history. *Pygmarrhopalites* are typically much smaller, less mobile, and have markedly shorter generation times compared to species of *Pogonognathellus*. Moore et al.'s (2005) study on cave Arrhopalitidae documented parthenogenesis and sexual maturation occurring as early as first instar in *Arrhopalites caecus* (Tullberg, 1871) from Wind Cave in South Dakota. Due to the absence of male *Pygmarrhopalites* A10 observed during our study, we cannot rule out asexuality. It is possible that parthenogenesis may have contributed to their lack of genetic structure between caves (after controlling for IBD). The ability for a single female to colonize new habitats without the need for males can facilitate dispersal to neighboring cave systems, possibly via small subterranean passages in the epikarst or fissures in bedrock during flooding events. However, male *Pygmarrhopalites* are usually present, but rarely encountered (Christiansen & Bellinger, 1996), and males have also been reported for *P. pavo*, a species that is morphologically similar to *Pygmarrhopalites* A10 (Christiansen & Bellinger, 1996).

### 4.2 Biogeography: evidence for vicariance across the Mississippi River Valley

The climatic and geological changes during the Pleistocene and their impacts on the distribution and diversity of North American cave fauna have been well documented (Porter, 2007). For example, the modern course of the Ohio River, formed by changing climate during the Pleistocene, bisects a major cave‐bearing karst region along the Indiana–Kentucky border. Niemiller, McCandless, et al. (2013) demonstrated that this river is a major biogeographic barrier, facilitating the divergence and subsequent isolation and speciation of troglobiotic cavefish populations. Like the Ohio River, the Mississippi River has also been implicated as a “hard” geographic barrier to dispersal for many surface species (e.g., Soltis, Morris, McLachlan, Manos, & Soltis, 2006), but its influence on the evolutionary history of cave‐dwelling organisms has yet to be evaluated, in part, because the geological history of the Mississippi River and its influence on regional cave‐bearing karst remain poorly understood.

Molecular divergence times of *Pygmarrhopalites* A10 populations spanning the Mississippi (Figures 6 and 7), patterns of genetic structure (Tables 4c, 5; Figure 5), and topology tests (Table 6) are consistent with the hypothesis that vicariance is the primary driver of genetic isolation in this species—providing prima facie evidence of vicariance across the Mississippi River for terrestrial cave arthropods, and accordingly, the first biogeographic evidence for the initial timing of Mississippi River entrenchment and bisection through Salem Plateau karst in Illinois and Missouri. An emerging chronological snapshot of ancestral Mississippi River geology coincides with our divergence time estimates. According to upland gravel distributions, the Mississippi River entrenched along its entire length during late Pliocene or early Pleistocene possibly due to a glacioeustatic lowering of sea level (Cupples & Van Arsdale, 2014). The combination of an increased late Pliocene Mississippi River discharge (Cox, Lumsden, & Van Arsdale, 2014) and subsequent Pleistocene glacial melt cycles beginning ~2.5 Ma (Balco & Rovey, 2008; Balco, Rovey, & Stone, 2005) could have facilitated valley growth and the erosion of karst habitat during the Pleistocene in this region. This scenario is further supported by the ages of burial sediments deposited in Mammoth Cave (Granger, Fabel, & Palmer, 2001), which provide a compelling parallel history of karst entrenchment by the Green River, a tributary within the Mississippi River watershed. Granger et al. (2001) attributed the oldest sediment deposit (~3.3 Ma) to a glacioeustatic lowering in sea level, immediately followed by a period of Green River excavation and bedrock incision lasting ~0.9 Ma. It is not unreasonable to consider that these climatic processes had the same effects on the geology of other karst regions occurring within the Mississippi River watershed (e.g., the Salem Plateau karst). Vicariance can also explain the strong patterns of isolation observed for the INC *Pygmarrhopalites* A10 populations (Figures 5, 6, 7), which are separated from other Illinois populations by the Kaskaskia River. Divergence time estimates between INC and other Illinois populations (~3.3 Ma) are similar to those estimated between Illinois and Missouri populations (~3.8 Ma), which makes sense given the Kaskaskia River is a large tributary of the Mississippi River: The karst entrenchment processes must have occurred at the same time as, or shortly after, this process took place for the Mississippi River.

The corroboration of timing information derived from both biological and geological data (Figure 7) supports the hypothesis that climatic and geological events beginning in the late Pliocene initiated and maintained genetic isolation between troglobiotic springtail populations in Illinois and Missouri, but the exact mode of gene flow across the preglacial Mississippi River and tributaries, prior to their genetic isolation, is not known. It is plausible that sections of karst were periodically isolated and rejoined by shifting meanders and periods of low flow, later removed by Plio‐Pleistocene entrenchment and excavation, providing intermittent subterranean passage for cave organisms until the late Pliocene or early Pleistocene.

The lack of genetic structure across the Mississippi River (Tables 4c and 5; Figure 5) and nonmonophyly (Table 6; Figure 6) for Illinois and Missouri populations of *Pogonognathellus* T4 cannot be explained by this scenario and is instead more consistent with a hypothesis of dispersal, rather than vicariance. Species in the genus *Pogonognathellus* are not ecologically restricted to caves and can maintain large surface populations; thus, they are unlikely to be affected by surface barriers to the same degree as obligate cave‐dwellers. Reports of springtails traveling vast distances via water surfaces (Coulson, Hodkinson, Webb, & Harrison, 2002), rafting (Hawes, Worland, Bale, & Convey, 2008), and air currents (e.g., Blackith & Disney, 1988; Coulson, Hodkinson, & Webb, 2003; Freeman, 1952; Hawes, Worland, Convey, & Bale, 2007) highlight a number of potential means for *Pogonognathellus* to passively disperse across the Mississippi River Valley that are typically unavailable to obligate subterranean springtails.

Their great abundance, low vagility, long‐term local persistence, and remarkable ecological specificity renders springtails excellent sources of information for inferring biogeographic processes (Garrick, Rowell, Simmons, Hillis, & Sunnucks, 2008). However, there are both technical and practical limitations to their use for biogeography, including but not restricted to (a) low single‐specimen DNA yields via modern extraction methodologies hinders opportunities for population‐level genomic‐scale analyses; (b) rampant cryptic speciation throughout Class Collembola, renders a priori taxonomic sampling for DNA extraction difficult, if not impossible; (c) limited taxonomic and gene coverage of available reference sequences—genomic datasets have only recently become available for three species (Faddeeva‐Vakhrusheva et al., 2016, 2017; Wu et al., 2017); and (d) the dearth of financial resources devoted to springtail research despite their ecological and evolutionary importance. These limitations are reflected in the small molecular datasets (relative to modern genomic standards) and reliance of mitochondrial markers for springtail phylogeography. Over‐reliance of mtDNA can produce misleading phylogenetic and biogeographic conclusions due to introgression, hybridization, paternal inheritance, and incomplete lineage sorting (Funk & Omland, 2003), but none of these processes have been reported for Collembola, except hybridization (Deharveng, Bedos, & Gisclard, 1998; Skarzynski, 2004). The development of more sensitive, nondestructive DNA extraction and genomic sequencing methods will certainly help alleviate these issues, improve the precision and accuracy of divergence time analyses, and bring springtail genetics into the big data era.

### 4.3 Cryptic diversity, short‐range endemism, and implications for conservation

Recent discoveries of cryptic species have challenged our current understanding of biological diversity (Fišer, Robinson, & Malard, 2018), and this paradigm shift is particularly evident in subterranean habitats where ideal conditions have fostered widespread cryptic speciation, including examples of recent divergence in cavefish (Niemiller, McCandless, et al., 2013), morphological stasis in amphipods (Trontelj et al., 2009), and morphological convergence in springtails (Christiansen, 1961). Therefore, it was important in this study to detect the presence of cryptic diversity and delimit OTUs prior to phylogeographic comparisons, to avoid interpreting interspecific variation as population‐level genetic structure. Large gaps in genetic distance frequencies (Figure 3) and the presence of strong interspecific genetic structure within caves (Table 2) provided evidence of highly divergent and sympatric lineages in cave samples. GMYC analysis identified 14 putative species within two morphospecies (10 *Pygmarrhopalites* and 4 *Pogonognathellus* OTUs), corroborated by the presence of large gaps between inter‐ and intra‐OTU distances for COI and 16S (Table 3; Figure 4). Lastly, minute differences in morphology among *Pygmarrhopalites* OTUs were also observed under compound light microscopy, providing additional support for the recognition of 10 distinct (and possibly new) species for this genus.

Only 38 species of *Pygmarrhopalites* and 11 species of *Pogonognathellus* are currently reported for all of North America (Christiansen & Bellinger, 1998; Felderhoff et al., 2010; Park, Bernard, & Moulton, 2011; Soto‐Adames & Taylor, 2013; Zeppelini & Christiansen, 2003; Zeppelini et al., 2009). Hence, the discovery of 14 putative species was surprising given the relatively small geographic scale of this study (~150 km stretch along the banks of the Mississippi River Valley). Molecular data also revealed that seven of the 10 *Pygmarrhopalites* OTUs may be single‐site endemics or have restricted ranges (Figure 6).

The detection of short‐range endemics, genetic isolation, and apparent cryptic diversity has major conservation implications. Reduced dispersal capacity observed for *Pygmarrhopalites* can increase their susceptibility to human disturbances such as land use practices, climate change, pollution, and invasive species—all of which pose major threats to fragile cave ecosystems (Culver & Pipan, 2009a; Taylor & Niemiller, 2016). In fact, growing concerns of karst groundwater contamination (Panno, Krapac, Weibel, & Bade, 1996) prompted *Pygmarrhopalites madonnensis* (Zeppelini & Christiansen, 2003), a troglobiotic springtail known from a single cave in Monroe Co., Illinois, to be listed as state endangered (Mankowski, 2010). This is concerning considering that our data indicate that single‐site endemics are not only extremely common but may also comprise a large majority of troglobiotic springtail diversity throughout this region. Lastly, unrecognized cryptic species complexes with allopatric ranges, presumed to be a single widely distributed species, may lead to misguided biodiversity conservation and management decisions.

## 5 CONCLUSIONS

Salem Plateau caves and their springtail inhabitants provide a model system for comparative phylogeographic studies addressing important questions in evolution and subterranean biogeography. We characterized and compared patterns of molecular diversity between species in the genera *Pygmarrhopalites* and *Pogonognathellus*, which led to three important findings. First, conflicting phylogeographic patterns between troglobiotic and eutroglophilic species distributed across the same geographic barriers suggests that different degrees of cave dependence can have major impacts on the dispersal capacity and genetic connectivity of cave organisms. Second, estimates of genetic structure and molecular divergence indicate that climatic and geological processes during the late Pliocene/early Pleistocene were major factors driving isolation between populations of troglobiotic cave organisms in Salem Plateau karst spanning the Mississippi River in Illinois and Missouri. Lastly, the large number of deeply divergent lineages and high rates of short‐range endemism detected in this study exposes a major knowledge gap in our understanding of cave microarthropod diversity and highlights potential conservation concerns under growing threats to cave biodiversity. Additional phylogeographic research and the development of genomic datasets for cave springtails will further contribute to our understanding of how and why organisms occupy, persist in, and adapt to cave environments—information critical for the development and implementation of conservation strategies needed to manage and protect cave biodiversity (Porter, 2007).

## AUTHOR CONTRIBUTIONS

A.D.K. contributed to research design, collected and analyzed data, and wrote the manuscript. S.J.T. conceived of the project, contributed to research design, provided access to cave sites, and assisted in data collection and manuscript writing. M.A.D. assisted in writing the manuscript and provided substantial molecular laboratory resources that contributed to data collection.

## DATA ACCESSIBILITY

All DNA sequence data from this study have been submitted to GenBank and are available under accession numbers MH269419–MH269696 and listed in Supporting information Appendix S1.
