# Supplementary material for: At the confluence of vicariance and dispersal: Phylogeography of cavernicolous springtails (Collembola: Arrhopalitidae, Tomoceridae) codistributed across a geologically complex karst landscape in Illinois and Missouri
Source: Ecol Evol. 2018 Sep 24;8(20):10306–25. doi: 10.1002/ece3.4507 (PMC6206200; doi:10.1002/ece3.4507)
Supplement: Supplementary file 1 [file ECE3-8-10306-s001.docx]

**Appendices**

**Appendix S1.** List of all taxa used in this study, including locality, sample information, and GenBank accession numbers for all sequences.

| Specimen ID^†^ | State | County | Cave^‡^ | Zone^§^ | Habitat^¶^ | COI | 16S | 28S D1-3 | 28S D7-10 | Histone-3 |
| --- | --- | --- | --- | --- | --- | --- | --- | --- | --- | --- |
| *Pygmarrhopalites* | |  |  |  |  |  |  |  |  |  |
| AVC_179IL1 | IL | Jackson | AVA | T | LL | MH269587 | MH269419 | MH269482 | MH269524 | MH269655 |
| BMC_091MO1 | MO | Perry | BMC | D | DP | MH269588 | MH269420 | MH269483 | MH269525 | MH269656 |
| BMC_091MO3 | MO | Perry | BMC | D | DP | MH269589 | MH269421 | MH269484 | MH269526 | MH269657 |
| BMC_091MO4 | MO | Perry | BMC | D | DP | MH269590 | — | MH269485 | MH269527 | MH269658 |
| BMC_093MO2 | MO | Perry | BMC | D | LL | MH269591 | MH269422 | MH269486 | MH269528 | MH269659 |
| BMC_097MO2 | MO | Perry | BMC | D | RS | MH269592 | MH269423 | MH269487 | MH269529 | MH269660 |
| CFC_108MO1 | MO | St. Louis | CFC | D | DP | MH269593 | MH269424 | MH269488 | MH269530 | MH269661 |
| ESC_084MO1 | MO | Ste. Genevieve | ESC | D | DP | MH269594 | MH269425 | MH269489 | MH269531 | MH269662 |
| FPC_027IL1 | IL | Monroe | FPC | D | LL | MH269595 | MH269426 | MH269490 | MH269532 | MH269663 |
| FPC_027IL2 | IL | Monroe | FPC | D | LL | MH269596 | MH269427 | MH269491 | MH269533 | MH269664 |
| FPC_027IL3 | IL | Monroe | FPC | D | LL | MH269597 | MH269428 | MH269492 | MH269534 | MH269665 |
| FPC_028IL1 | IL | Monroe | FPC | T | LL | MH269598 | MH269429 | MH269493 | MH269535 | MH269666 |
| FPC_029IL2 | IL | Monroe | FPC | E | LL | MH269599 | MH269430 | MH269494 | MH269536 | MH269667 |
| FPC_M10IL1 | IL | Monroe | FPC | D | LL | MH269600 | — | MH269495 | — | MH269668 |
| HSC_006IL1 | IL | Monroe | HSC | DT | LL | MH269601 | MH269431 | MH269496 | MH269537 | MH269669 |
| HSC_006IL2 | IL | Monroe | HSC | DT | LL | MH269602 | MH269432 | MH269497 | MH269538 | MH269670 |
| HSC_006IL3 | IL | Monroe | HSC | DT | LL | MH269603 | MH269433 | MH269498 | — | MH269671 |
| HSC_007IL2 | IL | Monroe | HSC | T | LL | MH269604 | MH269434 | MH269499 | MH269539 | MH269672 |
| ILC_193IL1 | IL | Monroe | ILC | T | LL | MH269605 | MH269435 | MH269500 | MH269540 | MH269673 |
| INC_158IL1 | IL | Randolph | INC | D | LL | MH269606 | MH269436 | MH269501 | MH269541 | MH269674 |
| INC_162IL1 | IL | Randolph | INC | D | SC | MH269607 | MH269437 | MH269502 | MH269542 | MH269675 |
| MYC_121MO1 | MO | Perry | MYC | D | LL | MH269608 | MH269438 | MH269503 | MH269543 | MH269676 |
| MYC_122MO2 | MO | Perry | MYC | D | SC | MH269609 | MH269439 | MH269504 | MH269544 | MH269677 |
| MYC_124MO2 | MO | Perry | MYC | D | DP | MH269610 | MH269440 | MH269505 | MH269545 | MH269678 |
| MYC_126MO2 | MO | Perry | MYC | D | LL | MH269611 | MH269441 | MH269506 | MH269546 | MH269679 |
| PAC_217IL1 | IL | Monroe | PAC | D | SC | MH269612 | MH269442 | MH269507 | MH269547 | MH269680 |
| PAC_217IL4 | IL | Monroe | PAC | D | SC | MH269613 | MH269443 | MH269508 | MH269548 | MH269681 |
| PAC_217IL5 | IL | Monroe | PAC | D | SC | MH269614 | MH269444 | MH269509 | MH269549 | MH269682 |
| PAC_217IL6 | IL | Monroe | PAC | D | SC | MH269615 | MH269445 | MH269510 | MH269550 | MH269683 |
| PSC_155IL1 | IL | Jackson | PSC | E | LL | MH269616 | MH269446 | MH269511 | MH269551 | MH269684 |
| SCC_143MO1 | MO | Perry | SCC | D | DP | MH269617 | MH269447 | MH269512 | MH269552 | MH269685 |
| SSC_141MO1 | MO | Perry | SSC | D | LL | MH269618 | MH269448 | MH269513 | MH269553 | MH269686 |
| SSC_141MO2 | MO | Perry | SSC | D | LL | MH269619 | MH269449 | MH269514 | MH269554 | MH269687 |
| SSC_141MO3 | MO | Perry | SSC | D | LL | MH269620 | MH269450 | MH269515 | MH269555 | MH269688 |
| SSC_142MO1 | MO | Perry | SSC | DT | LL | MH269621 | MH269451 | MH269516 | MH269556 | MH269689 |
| SSC_165MO1 | MO | Perry | SSC | D | GU | MH269622 | MH269452 | MH269517 | MH269557 | MH269690 |
| SSC_165MO2 | MO | Perry | SSC | D | GU | MH269623 | MH269453 | MH269518 | MH269558 | MH269691 |
| SSC_165MO3 | MO | Perry | SSC | D | GU | MH269624 | MH269454 | MH269519 | MH269559 | MH269692 |
| SSC_167MO1 | MO | Perry | SSC | D | LL | MH269625 | MH269455 | MH269520 | MH269560 | MH269693 |
| STC_199IL1 | IL | St. Clair | STC | D | DP | MH269626 | MH269456 | MH269521 | MH269561 | MH269694 |
| TMC_101MO2 | MO | Perry | TMC | D | RS | MH269627 | — | MH269522 | MH269562 | MH269695 |
| WRM_033IL1 | IL | Monroe | WRM | E | LL | MH269628 | MH269457 | MH269523 | MH269563 | MH269696 |
| *Pogonognathellus* | |  |  |  |  |  |  |  |  |  |
| AC1_133MO1 | MO | Cape Girardeau | AC1 | T | RS | MH269629 | MH269458 | — | MH269564 | — |
| AC3_134MO1 | MO | Cape Girardeau | AC3 | T | RS | MH269630 | MH269459 | — | MH269565 | — |
| AVC_171IL1 | IL | Jackson | AVA | T | LL | MH269631 | MH269460 | — | MH269566 | — |
| AVC_172IL1 | IL | Jackson | AVA | T | LL | MH269632 | — | — | — | — |
| AVC_173IL1 | IL | Jackson | AVA | D | LL | MH269633 | MH269461 | — | MH269567 | — |
| AVC_175IL2 | IL | Jackson | AVA | D | LL | MH269634 | MH269462 | — | MH269568 | — |
| AVC_179IL1 | IL | Jackson | AVA | T | LL | MH269635 | MH269463 | — | MH269569 | — |
| FPC_027IL1 | IL | Monroe | FPC | D | LL | MH269636 | MH269464 | — | MH269570 | — |
| FPC_027IL2 | IL | Monroe | FPC | D | LL | MH269637 | MH269465 | — | MH269571 | — |
| FPC_028IL1 | IL | Monroe | FPC | T | LL | MH269638 | MH269466 | — | MH269572 | — |
| FPC_029IL1 | IL | Monroe | FPC | E | LL | MH269639 | MH269467 | — | MH269573 | — |
| FPC_064IL1 | IL | Monroe | FPC | D | LL | MH269640 | MH269468 | — | MH269574 | — |
| FPC_068IL1 | IL | Monroe | FPC | D | LL | MH269641 | MH269469 | — | MH269575 | — |
| FPC_073IL1 | IL | Monroe | FPC | D | LL | MH269642 | MH269470 | — | MH269576 | — |
| FPC_073IL2 | IL | Monroe | FPC | D | LL | MH269643 | MH269471 | — | MH269577 | — |
| FPC_M20IL1 | IL | Monroe | FPC | D | LL | MH269644 | MH269472 | — | MH269578 | — |
| HSC_002IL1 | IL | Monroe | HSC | T | LL | MH269645 | MH269473 | — | — | — |
| HSC_005IL1 | IL | Monroe | HSC | T | LL | MH269646 | MH269474 | — | MH269579 | — |
| HSC_006IL1 | IL | Monroe | HSC | DT | LL | MH269647 | MH269475 | — | MH269580 | — |
| ILC_192IL1 | IL | Monroe | ILC | D | LL | MH269648 | MH269476 | — | MH269581 | — |
| ILC_193IL1 | IL | Monroe | ILC | T | LL | MH269649 | MH269477 | — | MH269582 | — |
| INC_163IL1 | IL | Randolph | INC | E | LL | MH269650 | MH269478 | — | MH269583 | — |
| MJP_013IL1 | IL | Monroe | MJP | T | LL | MH269651 | — | — | — | — |
| PAC_213IL1 | IL | Monroe | PAC | D | LL | MH269652 | MH269479 | — | MH269584 | — |
| PSC_155IL1 | IL | Jackson | PSC | E | LL | MH269653 | MH269480 | — | MH269585 | — |
| TMC_106MO1 | MO | Perry | TMC | D | LL | MH269654 | MH269481 | — | MH269586 | — |

^†^Sampled specimens for each genus are labeled according to Cave_SampleStateSpecimen.

^‡^Cave abbreviations are listed in Table 1.

^§^Zone abbreviations: E, entrance; T, twilight; DT, deep twilight; D, dark.

^¶^Habitat abbreviations: E, entrance; T, twilight; DT, deep twilight; D, dark. Habitat abbreviations: LL, leaf litter and other decaying plant material; DP, drip pools; RS, rock surfaces; SC, scat; GU, guano.

**Appendix S2.** PCR and sequencing primers, including a description of the PCR protocol and sequence alignment methods used in this study.

| Gene | Primer | Sequence (5'–3') | Length^†^ (bp) | Reference |
| --- | --- | --- | --- | --- |
| COI | jgLCO1490 | TITCIACIAAYCAYAARGAYATTGG | 597, 674 | Geller et al., 2013 |
|  | jgHCO2198 | TAIACYTCIGGRTGICCRAARAAYCA |  | Geller et al., 2013 |
|  |  |  |  |  |
| 16S | LR-J-12887M | CCGGTCTGAACTCAAATCATGT | 315, 528 | Zhang et al., 2014 |
|  | LR-N-12887M | CGACTGTTTAACAAAAACAT |  | Zhang et al., 2014 |
|  |  |  |  |  |
| 28S D1–3^‡^ | 28SrD1.2a | CCCSSGTAATTTAAGCATATTA | 1272 | Whiting, 2002 |
|  | 28Sbout | CCCACAGCGCCAGTTCTGCTTACC |  | Giribet et al., 2001 |
|  |  |  |  |  |
| 28S D7–10 | AS1 | CCGCAGCAGGTCTCCAAGGTGAA | 845, 857 | Xiong et al., 2008 |
|  | OP4 | CCGCCCCAGTCAAACTCCC |  | Xiong et al., 2008 |
|  |  |  |  |  |
| Histone-3^2^ | H3F1 | ATGGCTCGTACCAAGCAGACVGC | 329 | Colgan et al., 1998 |
|  | H3R2 | GTAACAATCATGCCCAAGGAYAT |  | Colgan et al., 1998 |

^†^Length of final alignment (*Pygmarrhopalites*, *Pogonognathellus*)

^‡^Due to inconsistent amplification, these loci were excluded for *Pogonognathellus*.

**PCR protocol**

Gene fragments were amplified by PCR with 12.5 μL of Go Taq Master Mix (Promega Corporation, Madison, WI), 8.5 μL of water, 1 μL of 10 μM forward and reverse primers and 2 μL of genomic DNA. Thermocycler settings for all primer combinations were as follows: 95°C 5 min; 40 cycles of 95°C 45 sec, 50°C 1.5 min, 72°C 1.5 min; and a final extension step for 72°C 10 min. Annealing temperature (50°C) was adjusted as needed for problematic amplifications. Successful amplification was verified via gel electrophoresis (90 V, 400 mA for 45 min) using a 1% agarose gel stained with GelRed (Biotium Inc., Hayward, CA). If multiple bands were observed, PCR was repeated with increasing annealing temperatures until single bands were obtained. Single-band PCR products were cleaned with Exo Sap-It Express (Affymetrix Inc., Santa Clara, CA) following manufactures protocol. Cleaned PCR products were pre-mixed with primers (1 μM) and sent to Eurofins Genomics LLC (Louisville, KY) for sequencing.

**Sequence alignment methods**

Forward and reverse sequences were assembled with Sequencher v. 5.4 (Gene Codes Corporation, Ann Arbor, MI). COI and H3 were aligned using the G-INSI-i alignment method in MAFFT v. 7.273 (Katoh & Standley, 2013) and translated to amino acids to check for stop codons. The GUIDANCE2 online server (Sela et al., 2015) was used to align 16S and 28S using MAFFT (max iterate=1000, 6mer pairwise alignment method, 100 bootstraps) to identify unreliably aligned positions for removal below default guidance score (cutoff=0.93).

**Appendix S3.** Outgroup taxa and GenBank sequence accession numbers for each locus included the (a) *Pygmarrhopalites* and (b) *Pogonognathellus* datasets.

| **Outgroups** | **COI** | **16S** | **28S D1-3** | **28S D7-10** | **Histone-3** |
| --- | --- | --- | --- | --- | --- |
| **(a)** |  |  |  |  |  |
| *Allacma fusca* | KT808323 | — | EU376054 | EU376054 | — |
| *Sminthurinus bimaculatus* | AY555545 | AY555555 | AF483398 | — | AY555566 |
| *Sminthurus viridis* | NC_010536 | NC_010536 | EF199973 | EF199973 | — |
| **(b)** |  |  |  |  |  |
| *Cryptopygus antarcticus* | NC_010533 | NC_010533 | — | EF199971 | — |
| *Folsomia candida* | KU198392 | KU198392 | — | JN981046 | — |
| *Lepidophorella* sp. | KJ716832 | — | — | — | — |

**Appendix S4.** The 95% HPD of site models selected by bModelTest for (**a**) the COI gene tree analyses used for GMYC species delimitation and (**b**) the rate-calibrated multilocus phylogenic analyses used for divergence time estimations. PS, posterior support for a model; CS, cumulative posterior support for a model; M, model number implemented in bModelTest. See Appendix in Bouckaert & Drummund (2017) for more information regarding specific model numbers.

| **(a)** | COI |  |  | **(b)** | 16S |  |  | COI |  | 28S D7-10 | | | 28S D1-3 | | | Histone-3 | | |
| --- | --- | --- | --- | --- | --- | --- | --- | --- | --- | --- | --- | --- | --- | --- | --- | --- | --- | --- |
| PS | CS | M |  | PS | CS | M | PS | CS | M | PS | CS | M | PS | CS | M | PS | CS | M |
| *Pygmarrhopalites* | | |  |  |  |  |  |  |  |  |  |  |  |  |  |  |  |  |
| 25.44% | 26.67% | 121123 |  | 13.33% | 13.33% | 123421 | 27.65% | 27.65% | 121123 | 11.48% | 11.48% | 123121 | 28.19% | 28.19% | 123123 | 22.08% | 22.08% | 121121 |
| 11.18% | 38.56% | 121323 |  | 12.58% | 25.91% | 123121 | 11.40% | 39.05% | 121323 | 10.34% | 21.82% | 123123 | 21.33% | 49.52% | 123423 | 8.07% | 30.15% | 121131 |
| 8.85% | 48.55% | 121134 |  | 7.73% | 33.65% | 123124 | 9.66% | 48.71% | 121134 | 9.46% | 31.27% | 121121 | 19.05% | 68.57% | 123124 | 6.55% | 36.70% | 123121 |
| 7.21% | 55.53% | 121324 |  | 6.07% | 39.71% | 123123 | 7.00% | 55.71% | 121324 | 7.06% | 38.33% | 123323 | 13.18% | 81.74% | 123425 | 6.45% | 43.15% | 123321 |
| 6.33% | 60.10% | 121131 |  | 5.57% | 45.28% | 121321 | 5.08% | 60.79% | 121121 | 5.46% | 43.79% | 123424 | 4.65% | 86.39% | 123143 | 6.19% | 49.33% | 123323 |
| 5.90% | 64.65% | 121343 |  | 5.41% | 50.69% | 123425 | 4.74% | 65.53% | 121131 | 5.34% | 49.12% | 123124 | 3.55% | 89.95% | 123453 | 5.81% | 55.15% | 121123 |
| 3.82% | 69.15% | 123124 |  | 4.84% | 55.53% | 123451 | 4.55% | 70.08% | 123124 | 5.06% | 54.19% | 123321 | 3.31% | 93.26% | 123421 | 5.37% | 60.52% | 121321 |
| 3.68% | 73.27% | 121121 |  | 4.61% | 60.14% | 123143 | 4.18% | 74.26% | 121343 | 4.41% | 58.60% | 121321 | 3.30% | 96.56% | 123145 | 4.86% | 65.37% | 123123 |
| 3.29% | 77.17% | 123324 |  | 4.51% | 64.65% | 123423 | 3.81% | 78.07% | 123324 | 4.29% | 62.88% | 123423 |  |  |  | 4.61% | 69.98% | 121323 |
| 2.25% | 79.53% | 121345 |  | 4.44% | 69.09% | 123141 | 2.17% | 80.24% | 121345 | 4.20% | 67.09% | 123421 |  |  |  | 2.34% | 72.33% | 123341 |
| 1.93% | 81.44% | 123145 |  | 4.25% | 73.34% | 123424 | 1.72% | 81.96% | 123141 | 3.11% | 70.20% | 121123 |  |  |  | 2.32% | 74.64% | 123141 |
| 1.84% | 83.31% | 123141 |  | 3.87% | 77.22% | 123145 | 1.66% | 83.62% | 123424 | 2.90% | 73.10% | 123141 |  |  |  | 2.25% | 76.90% | 123343 |
| 1.83% | 85.06% | 123424 |  | 2.66% | 79.88% | 121324 | 1.66% | 85.28% | 123121 | 2.86% | 75.96% | 121323 |  |  |  | 2.05% | 78.94% | 121341 |
| 1.73% | 86.74% | 123345 |  | 2.60% | 82.48% | 123453 | 1.56% | 86.84% | 123145 | 2.86% | 78.82% | 123324 |  |  |  | 2.05% | 80.99% | 121134 |
| 1.71% | 88.41% | 123341 |  | 2.31% | 84.79% | 121323 | 1.55% | 88.39% | 123345 | 2.60% | 81.41% | 123143 |  |  |  | 1.97% | 82.96% | 123324 |
| 1.65% | 90.03% | 123121 |  | 2.24% | 87.03% | 123456 | 1.51% | 89.91% | 123341 | 2.41% | 83.82% | 121131 |  |  |  | 1.93% | 84.89% | 123421 |
| 1.31% | 91.53% | 123321 |  | 1.94% | 88.97% | 121121 | 1.32% | 91.23% | 123321 | 2.34% | 86.17% | 123425 |  |  |  | 1.92% | 86.82% | 123124 |
| 1.17% | 92.65% | 123425 |  | 1.75% | 90.72% | 123454 | 1.11% | 92.34% | 121341 | 1.87% | 88.03% | 123343 |  |  |  | 1.75% | 88.57% | 123424 |
| 1.13% | 93.76% | 121341 |  | 1.71% | 92.43% | 121341 | 1.06% | 93.40% | 123425 | 1.47% | 89.51% | 123454 |  |  |  | 1.67% | 90.24% | 121343 |
| 1.11% | 94.86% | 121321 |  | 1.46% | 93.88% | 123321 | 1.05% | 94.45% | 121321 | 1.43% | 90.93% | 123145 |  |  |  | 1.67% | 91.91% | 123143 |
| 1.09% | 95.69% | 123454 |  | 1.15% | 95.04% | 123323 | 0.96% | 95.41% | 123323 | 1.32% | 92.26% | 123341 |  |  |  | 1.57% | 93.47% | 123423 |
| 1.06% |  |  |  |  |  |  |  |  |  | 1.27% | 93.53% | 121324 |  |  |  | 1.52% | 94.99% | 121324 |
|  |  |  |  |  |  |  |  |  |  | 1.15% | 94.68% | 123451 |  |  |  | 0.75% | 95.74% | 123451 |
|  |  |  |  |  |  |  |  |  |  | 1.11% | 95.79% | 121341 |  |  |  |  |  |  |
| *Pogonognathellus* | | |  |  |  |  |  |  |  |  |  |  |  |  |  |  |  |  |
| 31.25% | 31.25% | 123141 |  | 24.67% | 24.67% | 123124 | 34.26% | 34.26% | 123141 | 12.50% | 12.50% | 123421 |  |  |  |  |  |  |
| 21.69% | 52.93% | 123121 |  | 21.37% | 46.03% | 123145 | 15.82% | 50.08% | 123121 | 12.44% | 24.94% | 121323 |  |  |  |  |  |  |
| 10.42% | 63.35% | 123451 |  | 9.53% | 55.57% | 123425 | 13.28% | 63.36% | 123451 | 10.18% | 35.12% | 121324 |  |  |  |  |  |  |
| 7.55% | 70.89% | 123145 |  | 8.62% | 64.18% | 123424 | 8.45% | 71.81% | 123145 | 8.85% | 43.97% | 123424 |  |  |  |  |  |  |
| 7.33% | 78.22% | 123454 |  | 7.98% | 72.16% | 123456 | 8.21% | 80.01% | 123454 | 7.81% | 51.78% | 123121 |  |  |  |  |  |  |
| 7.12% | 85.34% | 123421 |  | 7.11% | 79.27% | 123454 | 6.34% | 86.36% | 123421 | 7.63% | 59.41% | 121321 |  |  |  |  |  |  |
| 5.06% | 90.40% | 123424 |  | 7.00% | 86.28% | 123121 | 4.09% | 90.44% | 123424 | 6.94% | 66.35% | 123425 |  |  |  |  |  |  |
| 5.06% | 95.46% | 123124 |  | 6.01% | 92.28% | 123141 | 3.58% | 94.03% | 123124 | 4.44% | 70.78% | 123451 |  |  |  |  |  |  |
|  |  |  |  | 4.20% | 96.49% | 123421 | 3.17% | 97.19% | 123456 | 4.21% | 74.99% | 121343 |  |  |  |  |  |  |
|  |  |  |  |  |  |  |  |  |  | 3.47% | 78.46% | 121345 |  |  |  |  |  |  |
|  |  |  |  |  |  |  |  |  |  | 3.04% | 81.50% | 123454 |  |  |  |  |  |  |
|  |  |  |  |  |  |  |  |  |  | 2.77% | 84.27% | 121341 |  |  |  |  |  |  |
|  |  |  |  |  |  |  |  |  |  | 2.69% | 86.96% | 123141 |  |  |  |  |  |  |
|  |  |  |  |  |  |  |  |  |  | 2.61% | 89.57% | 123423 |  |  |  |  |  |  |
|  |  |  |  |  |  |  |  |  |  | 2.52% | 92.08% | 123124 |  |  |  |  |  |  |
|  |  |  |  |  |  |  |  |  |  | 2.32% | 94.41% | 123456 |  |  |  |  |  |  |
|  |  |  |  |  |  |  |  |  |  | 1.00% | 95.41% | 123453 |  |  |  |  |  |  |

**Appendix S5.** Site model statistics for (**a**) the COI gene tree analyses used for GMYC species delimitation and (**b**) the rate-calibrated multilocus phylogenic analyses, selected by bModelTest.

| Site Partition | | Summary Statistic | BMT Model Indicator | BMT gamma Shape | BMT Proportion Invariable | has Gamma Rates | has Invariable Sites | Active Prop Invariable | Active Gamma Shape | has Equal Freqs |
| --- | --- | --- | --- | --- | --- | --- | --- | --- | --- | --- |
| **(a)** |  |  |  |  |  |  |  |  |  |  |
| *Pygmarrhopalites* COI | mean | 7.68 | 0.4791 | 0.2880 | 1 | 0.9981 | 0.2879 | 0.4791 | 0 |  |
|  | stderr | 0.03 | 0.0015 | 0.0008 |  | 0.0004 | 0.0008 | 0.0015 |  |  |
|  | stdev | 7.09 | 0.1170 | 0.0635 |  | 0.0438 | 0.0639 | 0.1170 |  |  |
|  | variance | 50.26 | 0.0137 | 0.0040 |  | 0.0019 | 0.0041 | 0.0137 |  |  |
|  | median | 6 | 0.4677 | 0.2938 |  | 1 | 0.2938 | 0.4677 |  |  |
|  | 95% HPD | 1–23 | 0.2505–0.7189 | 0.1597–0.4091 |  | 1–1 | 0.1597–0.4091 | 0.2505–0.7189 |  |  |
|  |  |  |  |  |  |  |  |  |  |  |
| *Pogonognathellus* COI | mean | 17.74 | 0.9482 | 0.4055 | 1 | 0.9573 | 0.4029 | 0.9482 | 0 |  |
|  | stderr | 0.03 | 0.0098 | 0.0034 |  | 0.0052 | 0.0037 | 0.0098 |  |  |
|  | stdev | 6.60 | 0.5460 | 0.1302 |  | 0.2022 | 0.1370 | 0.5460 |  |  |
|  | variance | 43.60 | 0.2981 | 0.0170 |  | 0.0409 | 0.0188 | 0.2981 |  |  |
|  | median | 14 | 0.8378 | 0.4431 |  | 1 | 0.4431 | 0.8378 |  |  |
|  | 95% HPD | 11–29 | 0.1793–1.9852 | 0.0804–0.5794 |  | 1–1 | 0–0.5457 | 0.1793–1.9852 |  |  |
| **(b)** |  |  |  |  |  |  |  |  |  |  |
| *Pygmarrhopalites* COI | mean | 7.76 | 0.4142 | 0.2666 | 1 | 0.9927 | 0.2661 | 0.4142 | 0 |  |
|  | stderr | 0.04 | 0.0012 | 0.0009 |  | 0.0012 | 0.0010 | 0.0012 |  |  |
|  | stdev | 7.03 | 0.0867 | 0.0623 |  | 0.0853 | 0.0640 | 0.0867 |  |  |
|  | variance | 49.36 | 0.0075 | 0.0039 |  | 0.0073 | 0.0041 | 0.0075 |  |  |
|  | median | 6 | 0.4123 | 0.2742 |  | 1 | 0.2742 | 0.4123 |  |  |
|  | 95% HPD | 1–23 | 0.2137– 0.5682 | 0.1305–0.3831 |  | 1–1 | 0.1297–0.3842 | 0.2137–0.5682 |  |  |
|  |  |  |  |  |  |  |  |  |  |  |
| *Pygmarrhopalites* 16S | mean | 16.67 | 0.6809 | 0.2428 | 1 | 0.8279 | 0.2315 | 0.6809 | 0 |  |
|  | stderr | 0.06 | 0.0070 | 2.83E-03 |  | 0.0068 | 0.0032 | 0.0070 |  |  |
|  | stdev | 7.77 | 0.3710 | 0.1337 |  | 0.3775 | 0.1484 | 0.3710 |  |  |
|  | variance | 60.42 | 0.1377 | 0.0179 |  | 0.1425 | 0.0220 | 0.1377 |  |  |
|  | median | 15 | 0.5944 | 0.2663 |  | 1 | 0.2661 | 0.5944 |  |  |
|  | 95% HPD | 5–30 | 0.2232–1.4050 | 0.0002–0.4260 |  | 0–1 | 0–0.4259 | 0.2232–1.4050 |  |  |
|  |  |  |  |  |  |  |  |  |  |  |
| *Pygmarrhopalites* 28S D1-3 | mean | 18.23 | 0.2302 | 0.4300 | 0.2397 | 0.9912 | 0.7378 | 0.2182 | 0.4066 |  |
|  | stderr | 0.04 | 0.0011 | 0.0134 | 0.0047 | 0.0022 | 0.0066 | 0.0051 | 0.0096 |  |
|  | stdev | 6.36 | 0.0985 | 0.4585 | 0.1786 | 0.0936 | 0.4398 | 0.1968 | 0.3978 |  |
|  | variance | 40.51 | 0.0097 | 0.2102 | 0.0319 | 0.0088 | 0.1935 | 0.0387 | 0.1582 |  |
|  | median | 15 | 0.2067 | 0.2779 | 0.2071 | 1 | 1 | 0.1950 | 0.2733 |  |
|  | 95% HPD | 12–28 | 0.0823–0.4460 | 0.0859–1.2548 | 0–0.5659 | 1–1 | 0–1 | 0–0.5659 | 0–1 |  |
|  |  |  |  |  |  |  |  |  |  |  |
| *Pygmarrhopalites* 28S D7-10 | mean | 13.81 | 0.3008 | 0.3569 | 0.9914 | 0.8878 | 0.3431 | 0.2820 | 0.9527 |  |
|  | stderr | 0.04 | 0.0112 | 0.0063 | 0.0017 | 0.0043 | 0.0067 | 0.0091 | 0.0131 |  |
|  | stdev | 7.93 | 0.4254 | 0.2163 | 0.0924 | 0.3156 | 0.2322 | 0.3697 | 0.2124 |  |
|  | variance | 62.96 | 0.1810 | 0.0468 | 0.0085 | 0.0996 | 0.0539 | 0.1367 | 0.0451 |  |
|  | median | 13 | 0.1738 | 0.3444 | 1 | 1 | 0.3437 | 0.1695 | 1 |  |
|  | 95% HPD | 1–26 | 0.0010–1.0265 | 0.0012–0.7078 | 1–1 | 0–1 | 0–0.7064 | 0–0.9446 | 1–1 |  |
|  |  |  |  |  |  |  |  |  |  |  |
| *Pygmarrhopalites* Hitone-3 | mean | 9.71 | 0.5719 | 0.3467 | 0.9988 | 0.8299 | 0.3339 | 0.5698 | 0.9979 |  |
|  | stderr | 0.04 | 0.0110 | 0.0053 | 0.0004 | 0.0070 | 0.0058 | 0.0109 | 0.0007 |  |
|  | stdev | 8.04 | 0.5224 | 0.2062 | 0.0345 | 0.3757 | 0.2227 | 0.5203 | 0.0462 |  |
|  | variance | 64.71 | 0.2729 | 0.0425 | 0.0012 | 0.1411 | 0.0496 | 0.2707 | 0.0021 |  |
|  | median | 8 | 0.3938 | 0.3864 | 1 | 1 | 0.3864 | 0.3926 | 1 |  |
|  | 95% HPD | 1–24 | 0.0990–1.6257 | 0–0.6238 | 1–1 | 0–1 | 0–0.6238 | 0.0968–1.6300 | 1–1 |  |
|  |  |  |  |  |  |  |  |  |  |  |
| *Pogonognathellus* COI | mean | 18.38 | 0.6127 | 0.3685 | 1 | 0.9781 | 0.3671 | 0.6127 | 0 |  |
|  | stderr | 0.04 | 0.0054 | 0.0023 |  | 0.0025 | 0.0024 | 0.0054 |  |  |
|  | stdev | 6.65 | 0.3087 | 0.1139 |  | 0.1462 | 0.1177 | 0.3087 |  |  |
|  | variance | 44.20 | 0.0953 | 0.0130 |  | 0.0214 | 0.0138 | 0.0953 |  |  |
|  | median | 14 | 0.5428 | 0.3886 |  | 1 | 0.3886 | 0.5428 |  |  |
|  | 95% HPD | 11–29 | 0.1793–1.2172 | 0.1325–0.5566 |  | 1–1 | 0.1273–0.5573 | 0.1793–1.2172 |  |  |
|  |  |  |  |  |  |  |  |  |  |  |
| *Pogonognathellus* 16S | mean | 19.23 | 0.5593 | 0.1219 | 0.9995 | 0.5457 | 0.0906 | 0.5578 | 0 |  |
|  | stderr | 0.04 | 0.0068 | 0.0022 | 0.0003 | 0.0069 | 0.0025 | 0.0066 |  |  |
|  | stdev | 6.64 | 0.3012 | 0.1010 | 0.0230 | 0.4979 | 0.1151 | 0.2965 |  |  |
|  | variance | 44.14 | 0.0907 | 0.0102 | 0.0005 | 0.2479 | 0.0132 | 0.0879 |  |  |
|  | median | 16 | 0.4640 | 0.0939 | 1 | 1 | 0.0256 | 0.4639 |  |  |
|  | 95% HPD | 11–30 | 0.2569–1.1272 | 0–0.3258 | 1–1 | 0–1 | 0–0.3256 | 0.2569–1.1272 |  |  |
|  |  |  |  |  |  |  |  |  |  |  |
| *Pogonognathellus* 28S D7-10 | mean | 15.42 | 0.4011 | 0.2757 | 0.9835 | 0.7210 | 0.2320 | 0.3755 | 0.9936 |  |
|  | stderr | 0.05 | 0.0074 | 0.0030 | 0.0015 | 0.0040 | 0.0034 | 0.0055 | 0.0023 |  |
|  | stdev | 8.88 | 0.4919 | 0.1941 | 0.1275 | 0.4485 | 0.2208 | 0.4488 | 0.0798 |  |
|  | variance | 78.81 | 0.2420 | 0.0377 | 0.0163 | 0.2011 | 0.0488 | 0.2014 | 0.0064 |  |
|  | median | 11 | 0.2507 | 0.2437 | 1 | 1 | 0.1898 | 0.2409 | 1 |  |
|  | 95% HPD | 5–29 | 0.0010–1.2880 | 0–0.6346 | 1–1 | 0–1 | 0–0.6344 | 0–1.1828 | 1–1 |  |
